# Biodiversity effects under climate extremes intensify with aridity in grasslands but not forests

**DOI:** 10.1038/s41559-026-03130-1

**Published:** 2026-07-15

**Authors:** Takehiro Sasaki, Daniela Hoss, Yuanyuan Huang, Yuki Iwachido, Liting Zheng, Luis Abdala-Roberts, Eric Allan, Harald Auge, Nadia Barsoum, Jürgen Bauhus, Christel Baum, Carl Beierkuhnlein, Friderike Beyer, Pedro Brancalion, Seraina L. Cappelli, Chengjin Chu, Dylan Craven, Caroline Daniel, Jiri Dolezal, Anne Ebeling, Marina Fagundes, Olga Ferlian, Gislene Ganade, Tobias Gebauer, Benoit Gendreau-Berthiaume, Douglas Godbold, Nathaly Guerrero-Ramírez, Joannès Guillemot, Peter Hajek, Andy Hector, Dirk Hölscher, Hervé Jactel, Anke Jentsch, Julia Koricheva, Holger Kreft, Juergen Kreyling, Vojtech Lanta, Jan Leps, Wei Lin, Maybeth Mendoza-Espinosa, Celine Meredieu, Christian Messier, Sebastian T. Meyer, Florencia Montagnini, Bart Muys, Charles Nock, Alain Paquette, William C. Parker, John D. Parker, Victor Parra-Tabla, John Parrotta, Gustavo B. Paterno, Noémie A. Pichon, Daniel Piotto, Wayne Polley, Quentin Ponette, Catherine Potvin, Peter B. Reich, Boris Rewald, Agnès Robin, Christiane Roscher, Hans Sanden, Michael Scherer-Lorenzen, Florian Schnabel, Eric B. Searle, Alrun Siebenkäs, Rachel J. Standish, David Tilman, Jasper van Ruijven, Kris Verheyen, Alexandra Weigelt, Martin Weih, Ramona Werner, Brian Wilsey, Bernhard Schmid, Forest Isbell, Nico Eisenhauer

**Affiliations:** 1 Graduate School of Environment and Information Sciences, Yokohama National University, Yokohama, Japan; 2 Institute for Multidisciplinary Sciences, Yokohama National University, Yokohama, Japan; 3 German Centre for Integrative Biodiversity Research (iDiv) Halle-Jena-Leipzig, Leipzig, Germany; 4 Institute of Biology, Leipzig University, Leipzig, Germany; 5 Tokyo Metropolitan Research Institute for Environmental Protection, Tokyo, Japan; 6 Universidad Nacional Autónoma de México, Escuela Nacional de Estudios Superiores-Unidad Mérida, Ucú, Mexico; 7 Institute of Plant Sciences, University of Bern, Bern, Switzerland; 8 Centre for Development and Environment, University of Bern, Bern, Switzerland; 9 Oeschger Centre for Climate Change, University of Bern, Bern, Switzerland; 10 Helmholtz Centre for Environmental Research—UFZ, Halle, Germany; 11 Forest Research, Alice Holt Lodge, Farnham, UK; 12 Chair of Silviculture, Faculty of Environment and Natural Resources, University of Freiburg, Freiburg, Germany; 13 Future Forests Cluster of Excellence, University of Freiburg, Freiburg, Germany; 14 Soil Science, Faculty of Agriculture, Civil and Environmental Engineering, University of Rostock, Rostock, Germany; 15 Biogeography, University of Bayreuth, Bayreuth, Germany; 16 Departamento de Botánica, University of Granada, Granada, Spain; 17 Department for Spatial Structures and Digitization of Forests, University of Göttingen, Göttingen, Germany; 18 Forest Science Department, University of São Paulo/ESALQ, Piracicaba, Brazil; 19 Center for Carbon Research on Tropical Agriculture, University of São Paulo, Piracicaba, Brazil; 20 re.green, Rio de Janeiro, Brazil; 21 WSL Institute for Snow and Avalanche Research SLF, Alpine Environment and Natural Hazards, Mountain Ecosystems, Davos, Switzerland; 22 State Key Laboratory of Biocontrol, School of Ecology, Shenzhen Campus of Sun Yat-sen University, Shenzhen, China; 23 GEMA Center for Genomics, Ecology & Environment, Universidad Mayor, Santiago, Chile, Facultad de Ciencias, Universidad Mayor, Santiago, Chile; 24 Data Observatory Foundation, Santiago, Chile; 25 Institute of Botany, Czech Academy of Sciences, Pruhonice, Czech Republic; 26 Department of Botany, Faculty of Science, University of South Bohemia, České Budějovice, Czech Republic; 27 Institute of Biodiversity, Ecology and Evolution, Friedrich Schiller University Jena, Jena, Germany; 28 Departamento de Ecologia, Universidade Federal do Rio Grande do Norte (UFRN), Natal, Brazil; 29 Geobotany, Faculty of Biology, and Future Forests Cluster of Excellence, University of Freiburg, Freiburg, Germany; 30 geo-konzept Society of Environmental Planning Systems mbH, Adelschlag, Germany; 31 Université du Québec à Montréal (UQAM) and en Outaouais (UQO), Quebec, Quebec Canada; 32 Department of Forest Protection and Wildlife Management, Faculty of Forestry and Wood Technology, Mendel University in Brno, Brno, Czech Republic; 33 Department of Ecosystem Management, Climate and Biodiversity, University of Natural Resources and Life Sciences (BOKU), Vienna, Austria; 34 Department of Biodiversity, Macroecology & Biogeography, University of Göttingen, Göttingen, Germany; 35 Centre of Biodiversity and Sustainable Land Use (CBL), University of Göttingen, Göttingen, Germany; 36 CIRAD, UMR Eco&Sols, Montpellier, France; 37 CIRAD, INRAE, Institut Agro, IRD, Eco&Sols, University of Montpellier, Montpellier, France; 38 Department of Biology and Leverhulme Centre for Nature Recovery, University of Oxford, Oxford, UK; 39 Tropical Silviculture and Forest Ecology, University of Göttingen, Göttingen, Germany; 40 INRAE, University of Bordeaux, Biogeco, Cestas, France; 41 Disturbance Ecology and Vegetation Dynamics, Bayreuth Center of Ecology and Environmental Research, University of Bayreuth, Bayreuth, Germany; 42 Department of Biological Sciences, Royal Holloway University of London, Egham, UK; 43 Campus Institute Data Science (CIDAS), University of Göttingen, Göttingen, Germany; 44 Experimental Plant Ecology, Institute of Botany and Landscape Ecology, University of Greifswald, Greifswald, Germany; 45 Universidad Autónoma de Yucatán, Merida, Mexico; 46 Terrestrial Ecology Research Group, TUM School of Life Sciences, Technical University of Munich, Munich, Germany; 47 Yale School of the Environment, New Haven, CT USA; 48 KU Leuven, Department of Earth & Environmental Sciences, Leuven, Belgium; 49 Department of Renewable Resources, Faculty of Agriculture, Life, and Environmental Sciences, University of Alberta, Edmonton, Alberta Canada; 50 Centre for Forest Research, Département des sciences biologiques, Université du Québec à Montréal, Montreal, Quebec Canada; 51 Ontario Ministry of Natural Resources, Sault Ste. Marie, Ontario Canada; 52 Smithsonian Environmental Research Center, Edgewater, MD USA; 53 Departamento de Ecologia Tropical, campus de Ciencias Biológicas y Agropecuarias, UADY, Merida, Mexico; 54 USDA Forest Service, International Institute of Tropical Forestry, Rio Piedras, PR USA; 55 WSL Swiss Federal Research Institute, Birmensdorf, Switzerland; 56 Centro de Formação em Ciências Agroflorestais, Universidade Federal do Sul da Bahia, Ilhéus, Brazil; 57 USDA, Agricultural Research Service, Temple, TX USA; 58 Earth and Life Institute, Université catholique de Louvain – UCLouvain, Louvain-la-Neuve, Belgium; 59 Department of Biology, McGill University, Montreal, Quebec Canada; 60 Smithsonian Tropical Research Institute, Balboa Ancon, Republic of Panama; 61 Institute for Global Change Biology and School for Environment and Sustainability, University of Michigan, Ann Arbor, MI USA; 62 Department of Forest Resources, University of Minnesota, Saint Paul, MN USA; 63 UFZ, Helmholtz Centre for Environmental Research, Physiological Diversity, Leipzig, Germany; 64 School of Environmental and Conservation Sciences, Murdoch University, Perth, Western Australia Australia; 65 Department of Ecology, Evolution and Behavior, University of Minnesota, Saint Paul, MN USA; 66 Bren School, University of California, Santa Barbara, CA USA; 67 Forest Ecology and Management group, Wageningen University & Research, Wageningen, the Netherlands; 68 Forest & Nature Lab, Department of Environment, Ghent University, Ghent, Belgium; 69 Department of Systematic Botany and Functional Biodiversity, Leipzig University, Leipzig, Germany; 70 Department of Crop Production Ecology, Swedish University of Agricultural Sciences, Uppsala, Sweden; 71 School of Integrative Plant Science, Cornell University, Ithaca, NY USA; 72 Department of Ecology, Evolution and Organismal Biology, Iowa State University, Ames, IA USA; 73 Remote Sensing Laboratories, Department of Geography, University of Zürich, Zurich, Switzerland

**Keywords:** Biodiversity, Climate-change ecology, Climate-change impacts

## Abstract

Biodiversity generally enhances ecosystem productivity, but whether such effects persist or intensify during climate extremes is unclear. Here we synthesized data from 75 biodiversity experiments across grasslands and forests to assess how aridity and soil nutrients modulate plant diversity effects under drought and heat extremes. Biodiversity most strongly enhanced productivity under extreme drought in more arid grasslands but had limited effects in forests under such conditions. More arid grasslands showed enhanced complementarity effects under extreme drought, whereas less arid grasslands favoured selection effects driven by productive species. Heat extremes did not produce comparable context-dependent changes in plant diversity effects across ecosystem types or aridity gradients. Soil nutrients did not have any detectable influence under either drought or heat extremes, suggesting that as climatic stress intensifies, hydric limitations override edaphic constraints. Our synthesis identifies when and where plant diversity most strongly enhances productivity, showing that its effects are the greatest under extreme drought in more arid grasslands.

## Abstract (web summary)

Biodiversity effects on ecosystem functioning under climate extremes may depend on ecosystem type and baseline aridity. Here the authors show that the effects of plant diversity on productivity are strongest under extreme drought in more arid grasslands, whereas comparable context dependence is not detected in forests or under heat extremes.

## Main

Plant diversity is known to enhance ecosystem productivity^1–6^; however, whether biodiversity effects on productivity intensify during climate extremes, and the environments and ecosystem types that show the strongest biodiversity effects have not been clarified^7^. Filling this knowledge gap has become increasingly urgent as droughts and heatwaves intensify worldwide^8–13^. Although biodiversity effects have been predicted to strengthen under environmental stress^14–18^, empirical evidence remains fragmented^19–24^. Previous studies have shown that biodiversity can enhance ecosystem resistance under climate extremes in global grasslands^13^ and promote ecosystem functioning under a range of experimental stressors across taxa^17,24–26^.

A key barrier to resolving how biodiversity–productivity relationships shift across environmental contexts during climate extremes is the restricted geographic and temporal scope of most experiments and observational datasets^13,27^. These constraints prevent a better understanding of the diverse environmental contexts and extreme climatic events that modulate biodiversity effects across sites. Recent global syntheses have facilitated important progress and shown that biodiversity effects on productivity increase with community age^25,28–30^ and are generally stronger in grassland and forest ecosystems than in agricultural or aquatic systems^29^. However, these syntheses have also reported relatively consistent biodiversity effects across spatial climate gradients^25,29^ and limited sensitivity to interannual climatic variability^28,31^. Thus, the contexts under which biodiversity can buffer productivity under climate extremes remain unresolved^7^.

Although global syntheses have frequently reported broadly consistent biodiversity effects across years and climates^5,25,28,29^, such aggregated patterns may obscure how biodiversity–productivity relationships respond to climate extremes and how the direction and magnitude of these responses depend on background environmental contexts, such as aridity and soil nutrients^13^. Observational studies show that the strength and even the direction of biodiversity effects can vary across time and among locations, including within the same ecosystem type or biome^20–22^. Indeed, global syntheses indicate that variations explained by random effects associated with the site or study can exceed the variations explained by fixed predictors of ecosystem functioning, such as species richness, functional diversity and community age^30,32^. This implies that local environmental conditions, such as baseline aridity and soil nutrients, may govern the extent to which biodiversity buffers productivity under climate extremes^26,30,33^. However, such contingencies remain largely unexplored, because most syntheses have focused on average biodiversity effects over time rather than on the responsiveness of these effects to climatic anomalies^13^. Without resolving these variations, biodiversity effects may be considered consistent across all environmental contexts, even under climate extremes. Here we move beyond previous work by explicitly testing how biodiversity effects on productivity are modulated by aridity, soil nutrients and ecosystem type (grasslands or forests) during climate extremes across a global compilation of biodiversity experiments. Thus, we aim to identify the environmental contexts represented in our dataset under which biodiversity most strongly supports ecosystem productivity under climate extremes. This not only advances the ecological theory of biodiversity–ecosystem functioning relationships but also provides actionable insights for building and prioritizing climate-resilient ecosystems^7,34^.

**Fig. 1:**
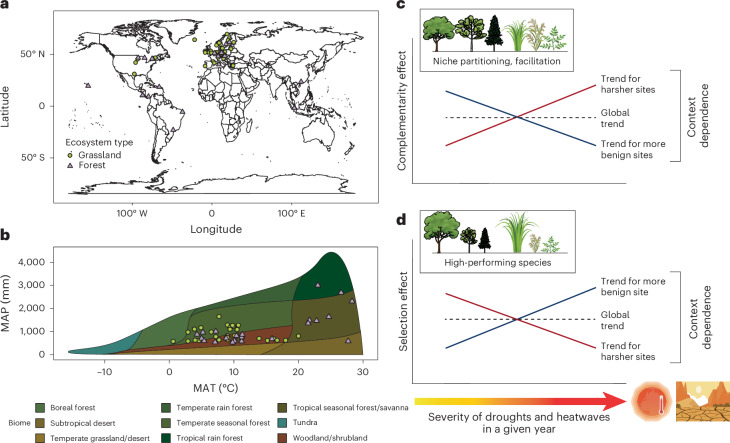
Geographic and climatic distribution of biodiversity experiments (*n* = 75) and conceptual framework used in this study. **a**, Points indicate the 51 grassland and 24 forest site locations. **b**, Experimental site distribution across mean annual temperature (MAT), mean annual precipitation (MAP) and Whittaker biomes. **c**,**d**, Conceptual frameworks illustrating how global gradients in aridity and soil nutrients are hypothesized to shape two key components of biodiversity effects (complementarity and selection effects) on productivity under climate extremes. Biodiversity effects appear globally consistent across years and climates (‘global trend’ in the panels) but may obscure large site-level variation in responses to interannual extreme climate conditions. In more arid or soil-nutrient-limited sites (harsher sites), complementarity effects are expected to strengthen during climate extremes, suggesting that niche partitioning and/or facilitation play a greater role under stress. In contrast, in less arid or soil-nutrient-rich sites (benign sites), climate extremes may amplify selection effects, potentially reflecting a stronger influence of dominant, high-performing species on ecosystem functioning. Illustrations created in the Mind the Graph platform.

**Extended Data Fig. 1:**
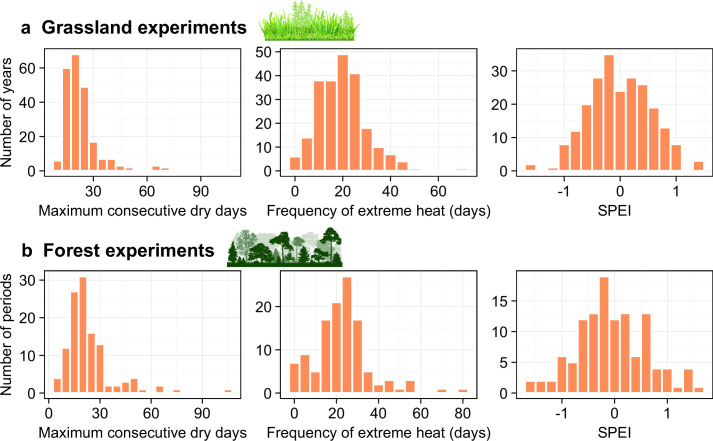
Frequency distribution of annual climate extreme indicators. The maximum consecutive dry days, frequency of extreme heat (the number of days exceeding the 95th percentile of daily maximum temperature), and annual mean of monthly Standardized Precipitation-Evapotranspiration Index (SPEI) values with a 3-month integration period for the grassland (**a**) and forest (**b**) experiments. At forest sites with multi-year measurement intervals, climate extreme indicators were averaged over each measurement period. Thus, the number of data points reflects the number of periods (that is, single years or multi-year intervals). Illustrations created in the Mind the Graph platform.

We synthesize data from globally distributed biodiversity experiments conducted in both grassland and forest ecosystems and collected over 2–23 years from 75 sites spanning broad gradients of aridity and soil nutrients (Fig. 1a,b and Supplementary Table 1). These comprehensive ecological datasets are integrated with long-term daily climate records to quantify interannual variability and extreme climatic conditions using three widely recognized indicators (Extended Data Fig. 1): (1) standardized precipitation–evapotranspiration index (SPEI), which integrates both precipitation and temperature to aggregate water balance over a short period, thereby capturing seasonal drought conditions (we calculate the monthly SPEI with a 3-month integration period and average this within a year; negative SPEI values indicate water deficiency)^35^; (2) maximum consecutive dry days, indicating the duration of the longest dry spell within a year (drought duration) and capturing meteorological drought extremes^36–38^; and (3) number of days exceeding the 95th percentile of daily maximum temperature, which reflects the frequency of extreme heat events (that is, heatwaves)^39^. We partition the net biodiversity effect into complementarity effects that arise from niche differentiation and/or facilitative interactions and selection effects that reflect the dominance of highly productive species^29,40^. We analyse the net biodiversity effect and its components in response to climate extremes using a metaregression model that includes both grassland and forest experiments. This model examines how biodiversity effects respond to climatic extremes and how these responses are influenced by interactions with ecosystem type, aridity index (precipitation/potential evapotranspiration, with lower values indicating higher aridity) and soil nutrients. We also include community age as a covariate because of its known influence on biodiversity–ecosystem functioning relationships^3,28–30^.

We hypothesize that complementarity effects become stronger during climate extremes in more arid or soil-nutrient-limited environments, where niche partitioning and/or facilitation are expected to enhance productivity under environmental stress (Fig. 1c)^14,16,26,30,41,42^. However, we also consider a contrasting possibility: unlike aridity-driven stress, severe soil nutrient limitation may instead reduce complementarity as a result of strong constraints on available niche spaces and environmental heterogeneity^43,44^. Selection effects are expected to strengthen under climate extremes in more mesic, nutrient-rich environments, where competitive dominance of high-performing species may drive ecosystem responses (Fig. 1d)^26,33,45^. Nonetheless, selection effects may also increase during climate extremes in arid environments, particularly when a few drought-tolerant species dominate community productivity under water-limited conditions^46,47^. To account for differences in productivity baselines across sites and years, which can influence the magnitude of biodiversity effects when measured on an absolute scale, we standardize all biodiversity effect metrics by the corresponding mean monoculture yield^26,29^. This approach captures variations in biodiversity effects on a relative scale and enables robust comparisons across environmental gradients over time and space.

## Results and discussion

### Overview of biodiversity effects across experimental sites

**Extended Data Fig. 2:**
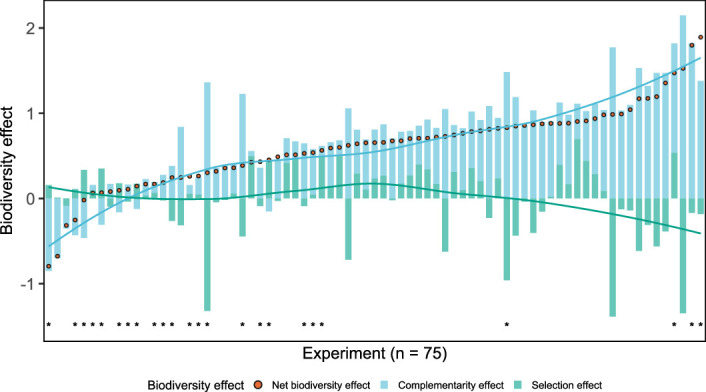
Overview of the biodiversity effects across experimental sites (*n* = 75). Biodiversity effects averaged across observation years for each experimental site are shown in ascending order of net biodiversity effects from left to right in the panel. Lines indicate locally estimated scatterplot smoothing fit. Asterisks indicate forest experiments. Biodiversity effect values are shown on the square-root-transformed scale (with original signs retained).

Across all experimental plots in grasslands and forests, complementarity effects accounted for a larger share of the net biodiversity effect than selection effects (Extended Data Fig. 2), which is consistent with previous global syntheses^28,29^. The net biodiversity effect and complementarity effect were generally positive, whereas the selection effect was either positive or negative depending on the experiment. Because dominant species can differ among environments, variation in selection effects among experiments may imply that biodiversity contributes to ecosystem functioning at broader spatial scales through spatial turnover in species performance^48^. Collectively, these results suggest that positive biodiversity–productivity relationships are more strongly associated with niche partitioning and/or facilitative interactions than with the dominance of a few high-performing species across grasslands and forests^29,40^. These findings provide a foundation for evaluating the sensitivity of biodiversity effects to climate extremes and for understanding how such responses interact with environmental contexts and ecosystem types. One important aspect of our study is the focus on biodiversity experiments with controlled species richness across diverse environmental contexts and climatic conditions, which minimizes any confounding effects from natural variation in community assembly^13,32^.

### Context-dependent biodiversity effects under extreme drought

**Fig. 2:**
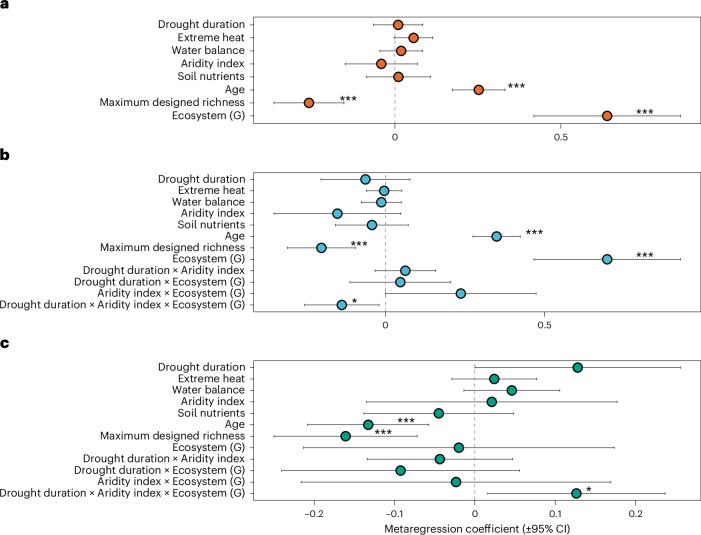
Modulation of biodiversity effects by climate extremes and environmental contexts. **a**–**c**, Effects of climate extreme indicators and their interactions with the aridity index, ecosystem type, community age, maximum designed species richness and soil nutrients on the net biodiversity effect (**a**), complementarity effect (**b**) and selection effect (**c**) across experiments (*n* = 75). The following climate extreme indicators were used: maximum consecutive dry days (drought duration), number of days exceeding the 95th percentile of daily maximum temperature (extreme heat) and annual mean of the monthly SPEI (water balance) in a given year. The metaregression models initially included the main effects of climate extreme indicators and their interactions with ecosystem type and site-level environmental conditions (aridity index and soil nutrients). Community age and maximum designed richness and their interactions with ecosystem type were also initially included as covariates. Non-significant interactions (*P* > 0.1) were subsequently removed to obtain the final parsimonious models. Effect of ecosystem type represents the difference between grassland (G) and forest, with forest serving as the reference level in the model. Soil nutrients were represented by the first principal component of soil properties (soil PC1; Extended Data Fig. 3). Points and error bars represent standardized fixed-effect coefficients and their 95% CIs in multilevel metaregression models. Statistical significance of fixed effects was assessed using two-sided tests with Knapp–Hartung adjustment. Asterisks indicate significance based on the model-derived *P* values (*P < 0.05; ** *P* < 0.01; *** *P* < 0.001). Exact coefficients, 95% CIs and exact *P* values are provided in Supplementary Table 2.

**Fig. 3:**
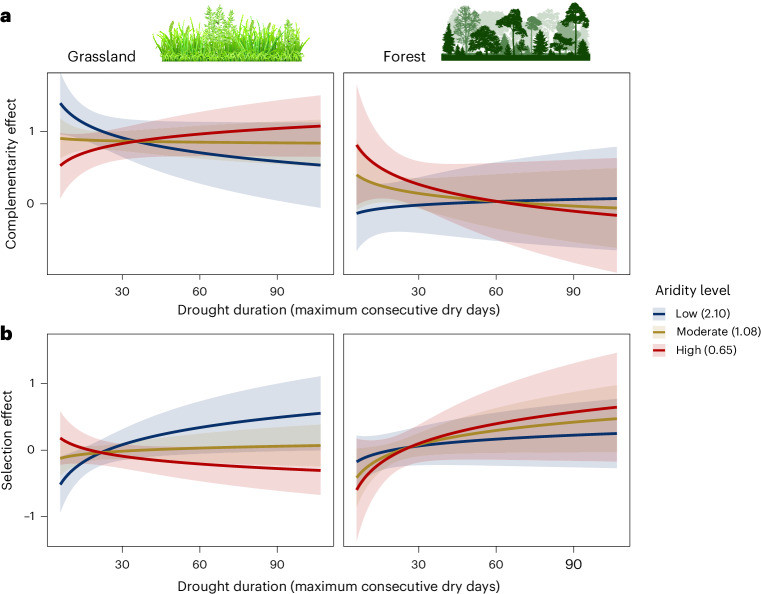
Interactive effects of drought duration, aridity and ecosystem type on complementarity and selection effects. **a**,**b**, Model-predicted relationships from multivariate metaregression analyses illustrating how drought duration (maximum consecutive dry days), aridity index and ecosystem type interactively influence complementarity (**a**) and selection (**b**) effects. For illustration and ease of visualization, representative values of the continuous aridity index (10th, 50th and 90th percentiles) were used to define high, moderate and low aridity levels (note that lower aridity index values indicate higher aridity). Lines represent fixed-effect model-predicted values, with other continuous predictors held at their mean values, and shaded areas represent 95% CIs for each aridity level. Curved relationships arise because drought duration was log_10_-transformed in the metaregression model, while predictions are plotted against its untransformed observed values. Predicted values of complementarity and selection effects are shown on the square-root-transformed scale (with original signs retained), which is consistent with the transformation used in the metaregression model. The interaction shown here was statistically significant in the multivariate metaregression analyses (Fig. 2 and Supplementary Table 2). Illustrations created in the Mind the Graph platform.

Biodiversity most strongly enhanced productivity in more arid grasslands during extreme drought. The metaregression model revealed a significant three-way interaction among maximum consecutive dry days, ecosystem type and site-level aridity index in explaining the complementarity effect (Figs. 2b and 3a and Supplementary Table 2). Post hoc simple-slope tests further showed that the maximum consecutive dry days × aridity index interaction was significant in grasslands (*β* = −0.07, 95% confidence interval (CI) [−0.14, −0.01], *P* = 0.03), but not in forests (*β* = 0.06, 95% CI [−0.03, 0.16], *P* = 0.20). This pattern supports our hypothesis that biodiversity buffering against drought extremes can intensify under water-limited conditions, where niche partitioning and/or facilitation may help sustain productivity as abiotic constraints strengthen^14,16,26,41,42^. Similarly, prior drought exposure can amplify complementarity during subsequent drought events^11,42,49^ and our results suggest that this insight may also apply to long-term exposure under naturally arid conditions. Selection effects, in contrast, increased with maximum consecutive dry days at less arid grassland sites (Figs. 2c and 3b and Supplementary Table 2). At these sites, drought extremes amplified selection effects while weakening complementarity, indicating that dominance by a few drought-tolerant species may play a disproportionate role in maintaining productivity under transient climatic stress^50^. However, at more arid grasslands, dominance by a few high-performing species became less effective under extreme drought. This is probably because severe moisture stress can increase the metabolic costs of maintaining physiological functions in these species, thereby reducing growth and elevating mortality risks^12,51^. The context-dependent biodiversity effect in grasslands was detected in response to increasing drought duration within a year, whereas it was not detected in response to the aggregated water balance, suggesting that acute indicators of drought extremes are more likely to reveal such context dependence than integrated drought metrics such as the SPEI^9,11^. Supplementary analyses using warm-season climate metrics also did not reveal any context-dependent effects of climate extremes on biodiversity effects. This suggests that biodiversity effects integrate climatic stress over annual timescales rather than being driven solely by within-season extremes (Supplementary Table 3)^52,53^. Aridity thus acts not only as an environmental filter on species performance but also as a modulator of the mechanisms through which biodiversity supports ecosystem functioning under climate extremes^12^. Recognizing such shifts is critical for predicting where and when biodiversity can buffer ecosystem productivity in an increasingly variable climate.

In contrast to grasslands, biodiversity effects in forests were not clearly modulated by site-level environmental context under extreme drought. The responses of complementarity and selection effects to increasing maximum consecutive dry days were not significantly influenced by interactions with site-level aridity conditions (Figs. 2b,c and 3 and Supplementary Table 2). This non-significant result should be interpreted cautiously, because the forest dataset included fewer experiments than the grassland dataset and therefore provided lower statistical power. Moreover, the slower demographic dynamics and structural inertia characteristic of forest ecosystems can obscure short-term biodiversity–functioning responses to climatic variability^31,54^. Forest stands dominated by long-lived species and characterized by low turnover rates may be less immediately responsive to interannual climatic extremes, with biodiversity effects potentially emerging only over longer timescales^31,54,55^. Drought impacts in trees may also be delayed across years and may be detected more sensitively in crown condition, tree health indicators or stable isotope measures than in annual growth^56–58^. However, the temporal resolution of the data for the forest experiments (maximum stand age of 23 years), which was often limited to multi-year intervals, prevented us from fully assessing short-term or lagged effects of climatic anomalies on biodiversity effects. Long-term, higher-frequency monitoring across a larger number of forest biodiversity experiments, ideally including more sensitive measures of tree performance, will therefore be needed to better resolve how climatic anomalies influence biodiversity effects in forests over longer timescales^31,55,59,60^.

### Limited roles of soil nutrients and heat extremes

**Extended Data Fig. 3:**
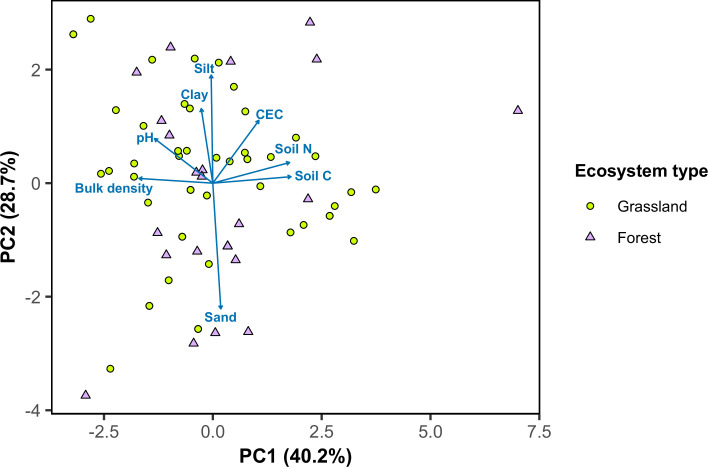
Principal component analysis biplots for the soil properties across experimental sites. Soil variables include soil organic carbon content (soil C), soil nitrogen content (soil N), soil pH (pH), sand, silt, and clay content, bulk density of the fine earth fraction, and cation exchange capacity (CEC). The percentages in the parentheses indicate the proportion of total variation explained by each dimension.

Across both grasslands and forests, soil nutrients exerted no detectable influence on biodiversity effects under climate extremes, because the interactions between soil nutrients (represented by the soil PC1; Extended Data Fig. 3) and climate extreme indicators were non-significant and therefore were excluded from the metaregression model (Fig. 2 and Supplementary Table 2). Although this result is tempered by the fact that the gridded global soil data used here may not fully represent local soil nutrient conditions at experimental sites, the absence of detectable soil–climate extreme interactions suggests that thermal and hydric limitations override edaphic effects on ecosystem productivity when climatic stress intensifies^61^. Under such conditions, water availability, not soil nutrient supply, becomes the dominant limiting factor for plant production, thereby lowering the relative importance of soil characteristics in modulating biodiversity–functioning relationships^61,62^. Aridity, rather than edaphic context, thus emerges as a key axis along which biodiversity modulates ecosystem functioning under extreme climatic events.

The frequency of extreme heat events showed no significant interaction with the site-level environmental context (aridity or soil nutrients) across grasslands and forests; therefore, these terms were removed during model simplification (Fig. 2 and Supplementary Table 2). It also did not generate consistent directional changes in net biodiversity, complementarity or selection effects. This result is consistent with the notion that biodiversity effects can help maintain ecosystem functioning under heat stress^25^. Thus, water limitation is a more proximate and pervasive constraint on primary productivity than heat alone^11,63,64^. Extreme heat typically reduces ecosystem functioning only when it coincides with low soil moisture and high atmospheric water demand, whereas heat events in the absence of severe hydric stress can have comparatively modest effects on biomass production^63–65^. Thus, although drought extremes can produce pronounced context dependence of biodiversity–productivity relationships in grasslands, many extreme heat events in our dataset may not have imposed sufficiently severe hydric stress to elicit comparable context-dependent responses.

### Community age and experimental design effects

Net biodiversity effects increased with community age across the ecosystem types (Fig. 2a). Moreover, complementarity effects strengthened while selection effects weakened with age (Fig. 2b,c), suggesting a general shift from dominance- to interaction-driven community productivity with time^3,28,29^. Gains in complementarity outweighed losses in selection, producing an overall enhancement of biodiversity effects^3,26,28^. Net biodiversity and complementarity effects were higher in grasslands than in forests, indicating stronger species interactions and a greater contribution of biodiversity to ecosystem functioning in grasslands^17^. This pattern could reflect greater resource limitations and strong belowground competition in grasslands compared with those in forests, with both limitations and competition enhancing niche differentiation and/or facilitative interactions among species^1,2^. Additionally, the smaller biodiversity effects observed in forests may partly reflect the shorter duration of many forest experiments, because tree-dominated communities generally require longer than herbaceous grasslands for biodiversity-driven differences in productivity to emerge^28,66^. The maximum designed richness was included as a covariate to account for differences in experimental design among studies. The negative coefficients of maximum designed richness should not be interpreted as evidence that biodiversity effects are intrinsically weaker in more biodiverse systems. Because our meta-analysis used only the highest-diversity treatment from each experiment, maximum designed richness functions here as an experiment-level design covariate describing the upper bound of richness manipulation, rather than a within-experiment test of richness effects. These coefficients therefore reflect among-experiment differences associated with richness design and cross-experiment standardization, not a biological decline in biodiversity effects with increasing richness itself.

## Conclusions

Our synthesis revealed that biodiversity most strongly enhanced ecosystem productivity under extreme drought in more arid grasslands but had a limited effect in forests under the same conditions. This observed contrast should be treated with caution, because the forest dataset included fewer experiments and was temporally coarser than the grassland dataset. Our results highlight the need to move beyond generalizations and towards tailored biodiversity-based strategies for climate adaptation. Grasslands in more arid regions may yield strong functional returns from conserving or restoring diversity because these systems are more likely to benefit from complementarity and/or facilitation under extreme drought. However, these inferences should be interpreted within the climatic and geographic range represented by the available experiments. Grassland sites were concentrated mainly in Europe and North America and did not fully capture the most arid grasslands, whereas Asian grasslands, tropical grasslands, dry tropical forests and Southern Hemisphere systems were sparsely represented or absent. Biodiversity effects may therefore respond nonlinearly under more extreme aridity, potentially weakening where strong abiotic filtering constrains niche partitioning and facilitation^67^; additional biodiversity experiments in these under-represented regions will be needed to test the generality of our findings. Expanded forest biodiversity experiments, longer time series and more sensitive measures of tree condition will likewise be important for determining whether biodiversity–climate interactions in forests emerge over longer timescales. Nonetheless, these findings show that the strength and mechanisms of biodiversity–productivity relationships can shift along aridity gradients and across ecosystem types represented here, reflecting systematic variation in how biodiversity influences ecosystem functioning under climatic extremes. Rather than assuming that biodiversity universally buffers environmental stress, our synthesis identifies the conditions under which it is most likely to have strong impacts, thereby offering a clear basis for targeted, nature-based strategies for climate adaptation.

## Methods

### Biodiversity experiment datasets

We assembled a global database by combining data from plant biodiversity experiments conducted in grasslands and forests. These experiments involved the manipulation of plant species richness and repeated measurements through time of species-level aboveground plant biomass (in grasslands) or basal area (in forests) within each experimental plot. Candidate datasets were identified from the authors’ knowledge of global grassland and forest biodiversity experiments, previous biodiversity–ecosystem functioning syntheses^13,28,30,32^ and associated research networks^68^. We used this approach because the present synthesis required raw data on monoculture and mixture productivity from repeated measurements through time, both to enable additive partitioning of biodiversity effects and to match their responses to climate extreme indicators over the corresponding observation intervals. Candidate datasets were then screened against the following inclusion criteria: (1) plant species richness was manipulated through sowing or planting; (2) experimental plots included both mixtures and corresponding monocultures to allow additive partitioning of biodiversity effects^40^; (3) raw time-series data on species-level aboveground biomass or basal area were available; and (4) the experiment spanned a minimum of 2 years for grasslands, with measurements performed in at least two separate years, and at least 3 years for forests, with measurements performed in at least three separate years, allowing for the calculation of two points of annual basal area increments.

In each experiment, to maintain the target species composition and diversity, non-target species were typically removed from the plots on a repeated basis. For the forest experiments, we excluded data from the first 2 years following the initial planting because tree-planting experiments typically require a period of establishment, as suggested by a previous synthesis^28^. In experiments that included additional treatments (for example, fertilization), we used only plots with ambient conditions that were not subjected to those treatments. In total, we used data from 51 grassland and 24 forest biodiversity experiments conducted worldwide and spanning 2 years to 23 years in duration (Fig. 1 and Supplementary Table 1). For each experiment, we first extracted data from plots with the maximum designed richness (that is, the highest manipulated species richness treatment in that experiment) and derived key variables for each year, including the biomass or basal area of individual species and community age (years since the start of the experiment). Then, for each experiment and year, we calculated the mean and variance of the net biodiversity, complementarity and selection effects (section ‘Additive partitioning of biodiversity effect’). This approach minimized within-experiment variation associated with different richness levels and avoided pseudo-replication in the subsequent metaregression analysis. The resulting meta-analytic dataset consisted of 340 independent data points, with 227 from grassland experiments and 113 from forest experiments.

### Annual aboveground productivity

For the grassland experiments, we used annual peak aboveground live biomass (g m^−2^) as a measure of annual aboveground productivity for each species in each plot and year^13,28^. These data were collected annually and consecutively across the grassland sites. For the forest experiments, we used annual basal area increment as an estimate of annual aboveground productivity for each species in each plot and year or multi-year period. Basal area (m^2^ ha^−1^) was calculated for each species using a standard equation based on the diameter at breast height^69^. For experiments where diameter at breast height measurements were unavailable, we estimated the basal area according to the basal diameter measured at ground level. Although this probably overestimated the basal area because basal diameters are generally larger than diameter at breast height as a result of natural stem taper, it should not have affected the relative temporal changes in species- or community-level basal area^28,30^. Although measurements were performed annually at some forest sites, surveys at multi-year intervals were performed at others. In such cases, we calculated the annual basal area increment by dividing the total change in basal area by the number of years between measurements. We assumed approximately linear growth during the measurement intervals, which is a common practice in long-term forest monitoring^28,30^.

### Additive partitioning of biodiversity effect

We calculated the net biodiversity effect, complementarity effect and selection effect, following the method of ref. ^40^. For each mixture plot, species-specific monoculture yields were taken from the corresponding monoculture plots in the same experiment and observation year or from the same observation interval in forest datasets with surveys at multi-year intervals. Where block structure was present, monoculture yields were matched to the block containing the focal mixture plot. The net biodiversity effect (NE) represents the deviation of yield (that is, annual aboveground productivity) in mixtures from the expected yield based on monocultures:

$${\rm{NE}}={Y}_{\rm{O}}-{Y}_{\rm{E}}$$

where *Y*_O_ is the observed yield in mixtures, which was calculated as the sum of the observed yields of all species in the mixture, and *Y*_E_ is the expected yield in mixtures, which was calculated as the sum of the monoculture yields of each species, with each multiplied by its initial sowing or planting proportion in the mixture.

The complementarity effect (CE) and selection effect (SE) were calculated as follows:

$${\rm{CE}}=N\times \overline{\Delta {\rm{RY}}}\times \overline{M}$$

$${\rm{SE}}=N\times \mathrm{cov}\left({\Delta {\rm{RY}}}_{i},{M}_{i}\right)$$

where *N* is the number of species in the mixture; ΔRY_*i*_ is the difference between the observed relative yield of species *i* in mixtures and its expected relative yield (that is, the proportion sown or planted); *M*_*i*_ is the monoculture yield of species *i*; ${\overline{\Delta{\rm{RY}}}}$ is the mean of ΔRY_*i*_ values across all species; $\bar{M}$ is the mean of *M*_*i*_ values across all species; and $\mathrm{cov}({\Delta {RY}}_{i},{M}_{i})$ is the covariance between the ΔRY_*i*_ and *M*_*i*_ values.

To account for variations in environmental conditions (for example, precipitation, temperature and soil nutrients) that can affect the magnitude of biodiversity effects across sites and years, we standardized all effect metrics by the corresponding mean monoculture yield^26,29^. This standardization makes the values unitless and enables consistent cross-site and cross-year comparisons. Subsequently, the biodiversity effect values were square-root-transformed to reduce skewness while retaining their original signs^40^.

### Climate datasets

We obtained daily precipitation (mm) and maximum temperature (°C) data for all experimental sites from 1982 to 2023 using the Global Historical Climatology Network daily database^70^. The year 1982 marks the earliest year with available data across all sites and the period until 2023 defines the baseline period for calculating an indicator of extreme heat events (details of which are provided in ‘Calculations of climate extreme indicators’)^8^. While the climate data uniformly cover this 1982–2023 period, the actual biodiversity observation periods varied by site and may have been shorter (Supplementary Table 1). To capture the aggregated water balance over relatively long periods and to represent seasonal drought conditions^35^, we extracted monthly SPEI values from SPEIbase^71^ v.2.10 using a 3-month integration period. To quantify the site-level aridity index (precipitation/potential evapotranspiration, unitless), we extracted monthly potential evapotranspiration from the Climatic Research Unit Gridded Time Series v.4.07 dataset^72^. The index was calculated as the mean annual ratio of precipitation to potential evapotranspiration over the period 2000–2020 and used as the baseline aridity for each site.

### Calculations of climate extreme indicators

The maximum consecutive dry days was calculated for each experimental site as the longest sequence of days with daily precipitation <1 mm in each year during the observational period. This metric was used as an annual indicator of extreme meteorological drought conditions^36,37^ and it has been widely used in climate science and global climate models, including Intergovernmental Panel on Climate Change assessments^38^. We confirmed that defining consecutive dry days using a 2-mm precipitation threshold^73^ produced nearly identical metaregression results (Supplementary Table 4), indicating that our findings were robust to the choice of dry-day threshold.

The number of days exceeding the 95th percentile of daily maximum temperature was also computed as an annual value for each site. The 95th percentile threshold was defined for each calendar month over the period 1982–2023 across the sites. For each year, we counted the number of days per month exceeding this threshold and the annual total was used to represent cumulative exposure to extreme heat^39,74^. In addition to event-based metrics such as the maximum consecutive dry days and frequency of extreme heat, we used the annual mean of the monthly SPEI with a 3-month integration period as a state-based indicator of seasonal hydroclimatic anomalies^35^. This value integrates both precipitation and temperature to quantify deviations from a relatively short-term climatic water balance, with negative values indicating drier-than-normal conditions^35^. Analyses based on the SPEI using 6- or 12-month integration periods yielded metaregression results nearly identical to those obtained with the 3-month SPEI (Supplementary Tables 5 and 6), further supporting the robustness of our conclusions to differences in the temporal integration window of the SPEI.

We thus used annual aggregates to quantify extreme drought, heat and water balance across all sites (Extended Data Fig. 1). This approach ensured comparability despite variations in biomass harvest timing and peak growing seasons among sites and interannual shifts in plant phenology. Extreme climatic events outside the core growing season, such as winter droughts or early-season heatwaves, can trigger physiological stress or alter soil moisture dynamics, ultimately affecting annual productivity. Aggregating climate variables at the annual scale thus captures the full spectrum of climatic stress experienced during the year of productivity measurement, which is consistent with recent global syntheses^52,53^. At some forest sites with multi-year measurement intervals, we averaged the maximum consecutive dry days, frequency of extreme heat and annual SPEI over the corresponding years. These interval-averaged climate metrics were then used to assess their effects on the annual basal area increment in the subsequent analyses. To evaluate the sensitivity of our results to the temporal window used to define climatic extremes, we recalculated these indicators for the warm season (April–September at all non-tropical sites, which were exclusively in the Northern Hemisphere and year-round in tropical regions) and repeated the metaregression analyses using these alternative metrics (Supplementary Table 3).

### Soil datasets

We used SoilGrids250m v.2.0 data^75^ to characterize the soil properties at each experimental site. Extracted variables included soil organic carbon content (g kg^−1^); soil nitrogen content (g kg^−1^); soil pH (in water); sand, silt and clay content (%); bulk density (kg m^−3^) of the fine earth fraction (<2 mm); and cation exchange capacity (cmol kg^−1^). To characterize site-level soil variations, we conducted a principal component analysis across all grassland and forest experiments using key soil variables. We retained the first principal component (soil PC1), which explained the largest proportion of total variance (40.2%) and captured a gradient of increasing soil carbon and nitrogen concentrations, thus reflecting soil nutrient conditions (Extended Data Fig. 3). We focused on soil PC1 because it best represented the gradient in soil nutrients that we hypothesized would modulate biodiversity effects under climate extremes (Fig. 1c,d).

### Data analysis

We used multilevel metaregression models to examine how climate extreme indicators (maximum consecutive dry days, frequency of extreme heat and SPEI), maximum designed richness and community age determine the net biodiversity effect, complementarity effect and selection effect over time across grassland and forest experimental sites. Each model initially included the main effects of climate extreme indicators and their interactions with ecosystem type and site-level environmental conditions (aridity index and soil PC1). Community age and maximum designed richness, along with their interactions with ecosystem type, were also included as covariates. Except for the standardized precipitation–evapotranspiration index, all explanatory variables were log_10_-transformed to reduce skewness. Subsequently, all explanatory variables were standardized to have zero mean and unit variance before analysis. To account for heterogeneity in the precision of effect-size estimates across sites, each observation was weighted by the inverse of its sampling variance, defined as the variance of the net biodiversity, complementarity and selection effects across plots at the maximum designed richness within a given year. Owing to multicollinearity (generalized variance inflation factor >3), the interactions between community age and ecosystem type and between maximum designed species richness and ecosystem type as well as the three-way interactions among climate extreme indicators, soil PC1 and ecosystem type were excluded from the model. All remaining main effects showed acceptable multicollinearity levels (generalized variance inflation factor of <3) and were therefore retained. In each model, non-significant interactions (*P* > 0.1) were subsequently removed to achieve a parsimonious model^13^. When significant three-way interactions were retained, we conducted post hoc simple-slope tests using linear contrasts within the fitted metaregression models to estimate and test ecosystem-specific slopes of the focal two-way interaction. Random effects were included in each model, thus representing a random intercept for the experimental site ID and another for the interaction between site ID and year. The latter accounted for temporal pseudo-replication by modelling the non-independence of repeated measurements within sites across years. All analyses were conducted in R v.4.4.1^76^, with metaregressions fitted using the metafor package^77^.

### Reporting summary

Further information on research design is available in the Nature Portfolio Reporting Summary linked to this article.

## Supplementary information

Supplementary Information Supplementary Tables 1–6.

Reporting Summary

Peer Review File

## Data Availability

The data needed to reproduce the results are available via figshare at 10.6084/m9.figshare.30850508 (ref. ^78^). Daily precipitation and maximum temperature data are available from the Global Historical Climatology Network daily database (https://www.ncei.noaa.gov/products/land-based-station/global-historical-climatology-network-daily). Monthly SPEI values are available from SPEIbase v.2.10 (https://developers.google.com/earth-engine/datasets/catalog/CSIC_SPEI_2_10#terms-of-use). Monthly potential evapotranspiration values are available from the Climatic Research Unit gridded Time Series 4.07 dataset (https://crudata.uea.ac.uk/cru/data/hrg/cru_ts_4.07/). Soil environmental data are available from the SoilGrids system (https://soilgrids.org/).

## Code availability

The codes needed to reproduce the results are available via figshare at 10.6084/m9.figshare.30850508 (ref. ^78^).
