# Supplementary material for: Biodiversity effects under climate extremes intensify with aridity in grasslands but not forests
Source: Nat Ecol Evol. 2026 Jul 15;10(8):1464–74. doi: 10.1038/s41559-026-03130-1 (PMC13441892; doi:10.1038/s41559-026-03130-1)
Supplement: Supplementary file 1 [file 41559_2026_3130_MOESM1_ESM.pdf]

nature ecology & evolution

Supplementary information

https://doi.org/10.1038/s41559-026-03130-1

Biodiversity effects under climate extremes intensify with aridity in grasslands but not forests

In the format provided by the authors and unedited

The PDF file includes:

Supplementary Tables 1 to 6

Supplementary Table 1. Characteristics of the grassland (*n* = 51) and forest (*n* = 24) sites in global biodiversity experiments.

Supplementary Table 2. Results of the multilevel meta-regression models for the effects of climate extreme indicators and their interactions with the aridity index, ecosystem type, community age, maximum designed species richness, and soil nutrients on the net biodiversity effect (a), complementarity effect (b), and selection effect (c) across experiments (*n* = 75).

Supplementary Table 3. Results of the multilevel meta-regression models for the effects of warm-season climate extreme indicators and their interactions with the aridity index, ecosystem type, community age, maximum designed species richness, and soil nutrients on the net biodiversity effect (a), complementarity effect (b), and selection effect (c) across experiments (*n* = 75).

Supplementary Table 4. Results of the multilevel meta-regression models for the effects of climate extreme indicators and their interactions with the aridity index, ecosystem type, community age, maximum designed species richness, and soil nutrients on the net biodiversity effect (a), complementarity effect (b), and selection effect (c) across experiments (*n* = 75).

Supplementary Table 5. Results of the multilevel meta-regression models for the effects of climate extreme indicators and their interactions with the aridity index, ecosystem type, community age, maximum designed species richness, and soil nutrients on the net biodiversity effect (a), complementarity effect (b), and selection effect (c) across experiments (*n* = 75).

Supplementary Table 6. Results of the multilevel meta-regression models for the effects of climate extreme indicators and their interactions with the aridity index, ecosystem type, community age, maximum designed species richness, and soil nutrients on the net biodiversity effect (a), complementarity effect (b), and selection effect (c) across experiments (*n* = 75).

Supplementary Table 1. Characteristics of the grassland (*n* = 51) and forest (*n* = 24) sites in global biodiversity experiments. Maximum designed richness (highest manipulated species richness level), aridity index (precipitation/potential evapotranspiration; unitless), MAP (mean annual precipitation; mm), and MAT (mean annual temperature; °C) at each site were calculated over the period 2000–2020.

Site	Lat	Long	Years	Study length	Maximum designed richness	Number of plots for the meta-level data	Aridity index	MAP	MAT
**Grasslands**									
Agrodiversity Belgium	50.98	3.82	2003–2005	3	4	32	1.02	748.1	11.3
Agrodiversity Canada	46.77	-71.20	2005–2007	3	4	32	1.70	1167.6	4.9
Agrodiversity Finland	61.67	27.21	2004–2005	2	4	32	1.24	613.8	4.6
Agrodiversity France	43.08	1.72	2004–2006	3	4	28	0.69	704.6	13.9
Agrodiversity Germany_a	48.77	9.18	2004–2006	3	4	22	1.15	827.0	10.5
Agrodiversity Germany_b	48.47	9.30	2005–2006	2	4	22	1.21	827.0	9.0
Agrodiversity Iceland_a	64.15	-21.75	2003–2005	3	4	32	2.59	1107.3	3.9
Agrodiversity Iceland_b	64.15	-21.75	2004–2006	3	4	22	2.59	1107.3	3.9
Agrodiversity Ireland_a	52.27	-6.50	2004–2006	3	4	22	1.87	1090.2	10.5

Agrodiversity Ireland_b	53.28	-8.73	2006–2007	2	4	32	2.10	1124.3	9.9
Agrodiversity Ireland_c	52.13	-8.27	2006–2007	2	4	32	2.14	1190.0	10.0
Agrodiversity Ireland_e	52.27	-6.50	2007–2009	3	4	22	1.87	1090.2	10.5
Agrodiversity Italy	40.74	8.54	2003–2005	3	4	22	0.63	686.8	16.2
Agrodiversity Lithuania_a	55.40	23.83	2003–2005	3	4	32	1.02	641.8	7.7
Agrodiversity Lithuania_b	55.40	23.83	2004–2006	3	4	32	1.02	641.8	7.7
Agrodiversity Lithuania_c	55.40	23.83	2004–2006	3	4	32	1.02	641.8	7.7
Agrodiversity Netherlands	51.96	5.64	2004–2006	3	4	32	1.08	748.0	10.9
Agrodiversity Norway_a	58.77	5.65	2004–2006	3	4	32	2.90	1652.8	7.7
Agrodiversity Norway_b	69.67	18.93	2003–2006	4	4	22	2.52	977.1	2.9
Agrodiversity Norway_c	59.67	10.85	2003–2005	3	4	22	1.59	984.2	6.5
Agrodiversity Norway_d	61.12	9.06	2004–2006	3	4	22	1.32	578.8	0.6
Agrodiversity Poland_a	52.43	16.30	2004–2006	3	4	22	0.78	564.2	10.1
Agrodiversity Poland_b	52.43	16.30	2005–2006	2	4	22	0.78	564.2	10.1
Agrodiversity Slovenia	46.05	14.47	2006–2007	2	4	32	1.81	1286.6	9.0
Agrodiversity Spain_a	41.73	2.88	2004–2006	3	4	22	0.58	615.2	15.2
Agrodiversity Sweden_a	55.92	13.12	2003–2005	3	4	32	1.12	697.9	8.8

Agrodiversity Sweden_b	55.92	13.12	2004–2006	3	4	32	1.12	697.9	8.8
Agrodiversity Sweden_c	65.32	21.39	2004–2006	3	4	22	1.33	587.3	2.9
Agrodiversity Switzerland	47.43	8.53	2003–2005	3	4	32	1.76	1181.6	9.4
Agrodiversity Wales_a	52.43	-4.02	2003–2006	4	4	22	2.29	1224.5	9.9
Agrodiversity Wales_b	51.95	-3.62	2004–2006	3	4	32	2.46	1270.2	9.8
BIODEPTH Germany	49.92	11.58	1996–1998	3	16	6	1.10	760.9	8.9
BIODEPTH Greece	39.14	26.52	1997–1999	3	18	8	0.49	626.6	16.7
BIODEPTH Ireland	51.78	-8.49	1996–1998	3	8	10	2.21	1278.0	10.7
BIODEPTH Portugal	38.77	-8.63	1997–1999	3	14	4	0.53	624.8	18.0
BIODEPTH Sheffield	53.20	-1.30	1996–1998	3	12	4	1.45	822.3	10.1
BIODEPTH Silwood	51.37	-0.62	1996–1998	3	11	8	1.12	695.9	10.8
BIODEPTH Sweden	63.75	20.28	1996–1998	3	12	4	1.42	631.2	4.0
BIODEPTH Switzerland	47.45	7.68	1995–1997	3	32	4	1.63	1099.2	9.4
CedarCreek BIOCON	45.40	-93.20	1998–2020	23	16	12	0.90	781.4	7.2
CedarCreek Biodiversity	45.40	-93.20	2001–2020	20	16	35	0.90	781.4	7.2
Czech	49.92	15.00	2003–2007	5	16	8	0.84	647.6	9.1
DivResource	51.39	11.88	2013–2018	6	4	4	0.75	533.7	9.8

EVENT	49.92	11.58	2005–2010	6	2	10	1.10	760.9	8.9
HectorUK	51.37	-0.62	1999–2001	3	27	4	1.12	695.9	10.8
Iowa	42.06	-95.82	2007–2009	3	4	33	0.76	722.5	9.2
Jena	50.95	11.62	2003–2020	18	60	4	1.09	745.2	9.0
PaNDiv	47.05	7.77	2017–2023	7	20	4	1.63	1099.2	9.4
Texas MEND	31.06	-97.35	2008–2015	8	9	16	0.55	809.5	20.0
Texas Richness-Evenness	31.06	-97.35	2001–2010	10	8	12	0.55	809.5	20.0
Wageningen	51.99	5.66	2000–2010	11	8	6	1.08	748.0	10.9
**Forests**									
BIOTREE-Kaltenborn	50.78	10.23	2011–2019	9	4	4	1.08	709.9	8.8
BrazilDry	-5.58	-36.95	2019–2023	5	16	15	0.36	585.0	27.7
B-Tree	48.32	16.07	2016–2022	7	4	4	0.69	579.6	11.2
CADE	23.51	111.81	2019–2022	4	8	16	1.49	1488.6	21.7
ECOLINK-Uppsala	59.82	17.67	2015–2021	7	4	3	0.93	539.2	7.1
EFForTS	-1.95	103.30	2015–2023	9	6	4	1.83	2310.3	28.4

FORBIO-Gedinne	50.00	4.98	2012–2023	12	4	10	1.13	818.2	10.4
FORBIO-Zedelgem	51.15	3.12	2012–2022	11	4	10	1.06	756.1	11.2
Hawaii	19.50	-155.25	1989–2002	14	2	20	1.20	1471.2	22.8
IDENT-Cloquet	46.68	-92.52	2013–2022	10	6	24	0.91	701.5	5.0
IDENT-Freiburg	48.02	7.83	2014–2020	7	6	24	1.17	871.8	11.3
IDENT-Macomer	40.24	8.72	2014–2022	9	6	18	0.65	686.8	15.9
IDENT-Montreal	45.42	-73.94	2011–2019	9	12	4	1.29	1009.9	7.2
IDENT-Outaouais	45.77	-75.01	2021–2023	3	4	20	1.35	966.9	5.2
IDENT-Sault-Ste-Marie	46.55	-84.46	2016–2023	8	6	8	1.39	876.2	4.3
Kreinitz	51.39	13.26	2008–2018	11	6	2	0.77	595.8	10.1
LaSelva1	10.43	-84.01	1992–1998	7	3	8	2.32	3002.1	23.0
LaSelva2	10.43	-84.01	1994–1998	5	4	8	2.32	3002.1	23.0
LaSelva3	10.43	-84.01	1994–1998	5	4	8	2.32	3002.1	23.0
MataDIV	-23.06	-48.65	2021–2023	3	20	3	1.24	1360.4	21.5
MyDiv	51.39	11.88	2016–2022	7	4	30	0.75	533.7	9.8
Sardinilla	9.32	-79.63	2006–2017	12	5	6	2.02	2685.0	26.6
Satakunta	61.72	21.98	2009–2016	8	5	5	1.15	597.0	5.4

ToaBaja	18.45	-66.17	1991–1993	3	2	9	1.20	1641.0	24.7

Supplementary Table 2. Results of the multilevel meta-regression models for the effects of climate extreme indicators and their interactions with the aridity index, ecosystem type, community age, maximum designed species richness, and soil nutrients on the net biodiversity effect (a), complementarity effect (b), and selection effect (c) across experiments (*n* = 75). The following climate extreme indicators were used: maximum consecutive dry days defined using a 1-mm daily precipitation threshold (drought duration), number of days exceeding the 95th percentile of daily maximum temperature (extreme heat), and annual mean of the monthly standardized precipitation evapotranspiration index calculated using a 3-month integration period (water balance) within a given year. Each model initially included the main effects of climate extreme indicators and their interactions with ecosystem type and site-level environmental conditions (aridity index and soil PC1). Community age and maximum designed richness, along with their interactions with ecosystem type, were also included as covariates. Non-significant interactions ($P > 0.1$) were subsequently removed to obtain the final parsimonious models. Effect of ecosystem type represents the difference between grassland (G) and forest ecosystems, with forest serving as the reference level in the model. Soil nutrients were represented by the first principal component of soil properties (soil PC1; fig. S2). Each subtable reports all fixed-effect terms retained in the final parsimonious model. All main effects and prespecified covariates were retained, whereas non-significant interactions were removed during model simplification. β, meta-regression coefficient; CI, confidence interval. Significant *P* values ($P < 0.05$) are in bold.

(a) ***Net biodiversity effect***

Term	β	95% CI	*P*

		(lower-upper)	
Drought duration	0.010	(-0.065, 0.084)	0.799
Extreme heat	0.056	(-0.002, 0.114)	0.059
Water balance	0.019	(-0.046, 0.083)	0.566
Aridity index	-0.041	(-0.150, 0.068)	0.460
Soil nutrients	0.011	(-0.087, 0.107)	0.832
Age	0.253	(0.173, 0.332)	**< 0.001**
Maximum designed richness	-0.259	(-0.365, -0.153)	**< 0.001**
Ecosystem (G)	0.639	(0.418, 0.861)	**< 0.001**

(b) ***Complementarity effect***

Term	β	95% CI (lower-upper)	*P*
Drought duration	-0.063	(-0.203, 0.078)	0.381
Extreme heat	-0.004	(-0.060, 0.052)	0.886
Water balance	-0.013	(-0.076, 0.050)	0.686
Aridity index	-0.151	(-0.351, 0.049)	0.139
Soil nutrients	-0.042	(-0.157, 0.072)	0.467
Age	0.349	(0.275, 0.424)	**<0.001**
Maximum designed richness	-0.201	(-0.308, -0.094)	**< 0.001**
Ecosystem (G)	0.697	(0.466, 0.928)	**< 0.001**
Drought duration × Aridity index	0.063	(-0.033, 0.158)	0.199
Drought duration × Ecosystem (G)	0.047	(-0.112, 0.206)	0.563
Aridity index × Ecosystem (G)	0.237	(-0.001, 0.474)	0.051
Drought duration × Aridity index × Ecosystem (G)	-0.137	(-0.255, -0.019)	**0.023**

(c) ***Selection effect***

Term	β	95% CI (lower-upper)	*P*

Drought duration	0.128	(-0.001, 0.257)	0.051
Extreme heat	0.024	(-0.029, 0.077)	0.369
Water balance	0.046	(-0.014, 0.106)	0.132
Aridity index	0.021	(-0.135, 0.178)	0.791
Soil nutrients	-0.045	(-0.138, 0.049)	0.346
Age	-0.133	(-0.209, -0.057)	**< 0.001**
Maximum designed richness	-0.161	(-0.250, -0.071)	**< 0.001**
Ecosystem (G)	-0.020	(-0.214, 0.174)	0.840
Drought duration × Aridity index	-0.044	(-0.134, 0.047)	0.346
Drought duration × Ecosystem (G)	-0.092	(-0.241, 0.056)	0.222
Aridity index × Ecosystem (G)	-0.023	(-0.217, 0.170)	0.812
Drought duration × Aridity index × Ecosystem (G)	0.126	(0.016, 0.237)	**0.025**

Supplementary Table 3. Results of the multilevel meta-regression models for the effects of warm-season climate extreme indicators and their interactions with the aridity index, ecosystem type, community age, maximum designed species richness, and soil nutrients on the net biodiversity effect (a), complementarity effect (b), and selection effect (c) across experiments (*n* = 75). The following climate extreme indicators were used: maximum consecutive dry days defined using a 1-mm daily precipitation threshold (drought duration), number of days exceeding the 95th percentile of daily maximum temperature (extreme heat), and mean of the monthly standardized precipitation evapotranspiration index calculated using a 3-month integration period (water balance) during the warm season. The warm season was defined as April–September for all non-tropical sites, which were exclusively located in the Northern Hemisphere, and year-round for tropical regions. Each model initially included the main effects of climate extreme indicators and their interactions with ecosystem type and site-level environmental conditions (aridity index and soil PC1). Community age and maximum designed richness, along with their interactions with ecosystem type, were also included as covariates. Non-significant interactions ($P > 0.1$) were subsequently removed to obtain the final parsimonious models. Effect of ecosystem type represents the difference between grassland (G) and forest ecosystems, with forest serving as the reference level in the model. Soil nutrients were represented by the first principal component of soil properties (soil PC1; fig. S2). Each subtable reports all fixed-effect terms retained in the final parsimonious model. All main effects and prespecified covariates were retained, whereas non-significant interactions were removed during model simplification. β, meta-regression coefficient; CI, confidence interval. Significant *P* values ($P < 0.05$) are in bold. In contrast to the models based

on annual climate aggregates (Supplementary Table 2), these warm-season models did not detect context-dependent effects of climate extremes on biodiversity effects.

(a) ***Net biodiversity effect***

Term	β	95% CI (lower-upper)	*P*
Drought duration	0.023	(-0.050, 0.096)	0.541
Extreme heat	0.037	(-0.021, 0.096)	0.210
Water balance	0.017	(-0.045, 0.080)	0.583
Aridity index	-0.041	(-0.151, 0.070)	0.467
Soil nutrients	0.014	(-0.083, 0.112)	0.778
Age	0.259	(0.180, 0.339)	**< 0.001**
Maximum designed richness	-0.258	(-0.365, -0.151)	**< 0.001**
Ecosystem (G)	0.661	(0.436, 0.886)	**< 0.001**

(b) ***Complementarity effect***

Term	β	95% CI (lower-upper)	*P*
Drought duration	0.006	(-0.061, 0.074)	0.856
Extreme heat	-0.026	(-0.079, 0.027)	0.336
Water balance	-0.034	(-0.093, 0.025)	0.255
Aridity index	-0.108	(-0.299, 0.082)	0.265
Soil nutrients	-0.038	(-0.153, 0.077)	0.518
Age	0.348	(0.275, 0.420)	**< 0.001**
Maximum designed richness	-0.196	(-0.304, -0.088)	**< 0.001**
Ecosystem (G)	0.741	(0.512, 0.970)	**< 0.001**
Water balance × Soil nutrients	0.051	(-0.007, 0.108)	0.085
Aridity index × Ecosystem (G)	0.189	(-0.035, 0.412)	0.097

**(c)** ***Selection effect***			
Term	β	95% CI (lower-upper)	*P*
Drought duration	0.014	(-0.049, 0.077)	0.652
Extreme heat	0.035	(-0.018, 0.088)	0.196
Water balance	0.060	(0.001, 0.120)	**0.046**
Aridity index	-0.001	(-0.096, 0.095)	0.990
Soil nutrients	-0.040	(-0.130, 0.051)	0.389
Age	-0.120	(-0.195, -0.045)	**0.002**
Maximum designed richness	-0.167	(-0.259, -0.076)	**< 0.001**
Ecosystem (G)	-0.031	(-0.226, 0.163)	0.751

Supplementary Table 4. Results of the multilevel meta-regression models for the effects of climate extreme indicators and their interactions with the aridity index, ecosystem type, community age, maximum designed species richness, and soil nutrients on the net biodiversity effect (a), complementarity effect (b), and selection effect (c) across experiments (*n* = 75). The following climate extreme indicators were used: maximum consecutive dry days defined using a 2-mm daily precipitation threshold (drought duration), number of days exceeding the 95th percentile of daily maximum temperature (extreme heat), and annual mean of the monthly standardized precipitation evapotranspiration index calculated using a 3-month integration period (water balance) within a given year. Each model initially included the main effects of climate extreme indicators and their interactions with ecosystem type and site-level environmental conditions (aridity index and soil PC1). Community age and maximum designed richness, along with their interactions with ecosystem type, were also included as covariates. Non-significant interactions ($P > 0.1$) were subsequently removed to obtain the final parsimonious models. Effect of ecosystem type represents the difference between grassland (G) and forest ecosystems, with forest serving as the reference level in the model. Soil nutrients were represented by the first principal component of soil properties (soil PC1; fig. S2). Each subtable reports all fixed-effect terms retained in the final parsimonious model. All main effects and prespecified covariates were retained, whereas non-significant interactions were removed during model simplification. β, meta-regression coefficient; CI, confidence interval. Significant *P* values ($P < 0.05$) are in bold. Results were nearly identical to those obtained when the models used maximum consecutive dry days defined with a 1-mm precipitation threshold (Supplementary Table 2).

(a) ***Net biodiversity effect***			
Term	β	95% CI (lower-upper)	*P*
Drought duration	0.026	(-0.049, 0.101)	0.500
Extreme heat	0.056	(-0.001, 0.113)	0.055
Water balance	0.023	(-0.042, 0.088)	0.479
Aridity index	-0.035	(-0.144, 0.075)	0.534
Soil nutrients	0.010	(-0.087, 0.107)	0.841
Age	0.251	(0.172, 0.331)	**< 0.001**
Maximum designed richness	-0.258	(-0.364, -0.153)	**< 0.001**
Ecosystem (G)	0.638	(0.417, 0.860)	**< 0.001**
(b) ***Complementarity effect***			
Term	β	95% CI (lower-upper)	*P*
Drought duration	-0.008	(-0.136, 0.120)	0.902
Extreme heat	-0.005	(-0.060, 0.050)	0.870
Water balance	0.003	(-0.060, 0.066)	0.919
Aridity index	-0.141	(-0.339, 0.057)	0.161
Soil nutrients	-0.042	(-0.157, 0.074)	0.479
Age	0.344	(0.270, 0.419)	**< 0.001**
Maximum designed richness	-0.201	(-0.309, -0.094)	**< 0.001**
Ecosystem (G)	0.691	(0.459, 0.923)	**< 0.001**
Drought duration × Aridity index	0.061	(-0.034, 0.155)	0.209
Drought duration × Ecosystem (G)	0.034	(-0.115, 0.182)	0.657
Aridity index × Ecosystem (G)	0.231	(-0.006, 0.467)	0.056
Drought duration × Aridity index × Ecosystem (G)	-0.138	(-0.257, -0.018)	**0.024**
(c) ***Selection effect***			

Term	β	95% CI (lower-upper)	*P*
Drought duration	0.121	(0.005, 0.238)	**0.042**
Extreme heat	0.029	(-0.023, 0.081)	0.272
Water balance	0.043	(-0.018, 0.103)	0.164
Aridity index	0.019	(-0.138, 0.175)	0.815
Soil nutrients	-0.047	(-0.141, 0.048)	0.333
Age	-0.132	(-0.208, -0.056)	**< 0.001**
Maximum designed richness	-0.159	(-0.248, -0.069)	**< 0.001**
Ecosystem (G)	-0.013	(-0.209, 0.183)	0.899
Drought duration × Aridity index	-0.043	(-0.132, 0.045)	0.337
Drought duration × Ecosystem (G)	-0.105	(-0.246, 0.037)	0.147
Aridity index × Ecosystem (G)	-0.012	(-0.207, 0.182)	0.901
Drought duration × Aridity index × Ecosystem (G)	0.128	(0.016, 0.241)	**0.026**

Supplementary Table 5. Results of the multilevel meta-regression models for the effects of climate extreme indicators and their interactions with the aridity index, ecosystem type, community age, maximum designed species richness, and soil nutrients on the net biodiversity effect (a), complementarity effect (b), and selection effect (c) across experiments (*n* = 75). The following climate extreme indicators were used: maximum consecutive dry days defined using a 1-mm daily precipitation threshold (drought duration), number of days exceeding the 95th percentile of daily maximum temperature (extreme heat), and annual mean of the monthly standardized precipitation evapotranspiration index calculated using a 6-month integration period (water balance) within a given year. Each model initially included the main effects of climate extreme indicators and their interactions with ecosystem type and site-level environmental conditions (aridity index and soil PC1). Community age and maximum designed richness, along with their interactions with ecosystem type, were also included as covariates. Non-significant interactions (P > 0.1) were subsequently removed to obtain the final parsimonious models. Effect of ecosystem type represents the difference between grassland (G) and forest ecosystems, with forest serving as the reference level in the model. Soil nutrients were represented by the first principal component of soil properties (soil PC1; fig. S2). Each subtable reports all fixed-effect terms retained in the final parsimonious model. All main effects and prespecified covariates were retained, whereas non-significant interactions were removed during model simplification. β, meta-regression coefficient; CI, confidence interval. Significant P values (P < 0.05) are in bold. Analyses using the 6-month SPEI produced meta-regression results nearly identical to those obtained with the 3-month SPEI used in the main models (Supplementary Table 2).

(a) *Net biodiversity effect*

Term	β	95% CI (lower-upper)	*P*
Drought duration	0.009	(-0.065, 0.084)	0.802
Extreme heat	0.056	(-0.002, 0.114)	0.059
Water balance	0.020	(-0.045, 0.084)	0.554
Aridity index	-0.041	(-0.150, 0.068)	0.460
Soil nutrients	0.011	(-0.086, 0.108)	0.827
Age	0.253	(0.174, 0.332)	**< 0.001**
Maximum designed richness	-0.259	(-0.365, -0.153)	**< 0.001**
Ecosystem (G)	0.639	(0.418, 0.861)	**< 0.001**

(b) *Complementarity effect*

Term	β	95% CI (lower-upper)	*P*
Drought duration	-0.066	(-0.206, 0.075)	0.359
Extreme heat	-0.005	(-0.061, 0.050)	0.849
Water balance	-0.022	(-0.086, 0.041)	0.493
Aridity index	-0.150	(-0.350, 0.051)	0.143
Soil nutrients	-0.040	(-0.155, 0.075)	0.493
Age	0.349	(0.274, 0.424)	**< 0.001**
Maximum designed richness	-0.201	(-0.308, -0.094)	**< 0.001**
Ecosystem (G)	0.697	(0.466, 0.929)	**< 0.001**
Drought duration × Aridity index	0.063	(-0.033, 0.158)	0.199
Drought duration × Ecosystem (G)	0.048	(-0.111, 0.207)	0.555
Aridity index × Ecosystem (G)	0.234	(-0.005, 0.472)	0.055
Drought duration × Aridity index × Ecosystem (G)	-0.138	(-0.256, -0.020)	**0.022**

(c) *Selection effect*

Term	β	95% CI (lower-upper)	*P*
Drought duration	0.127	(-0.001, 0.256)	0.052
Extreme heat	0.024	(-0.028, 0.077)	0.365
Water balance	0.049	(-0.011, 0.109)	0.110
Aridity index	0.019	(-0.138, 0.177)	0.810
Soil nutrients	-0.045	(-0.139, 0.049)	0.348
Age	-0.130	(-0.206, -0.054)	**< 0.001**
Maximum designed richness	-0.161	(-0.251, -0.071)	**< 0.001**
Ecosystem (G)	-0.019	(-0.214, 0.176)	0.846
Drought duration × Aridity index	-0.043	(-0.133, 0.048)	0.355
Drought duration × Ecosystem (G)	-0.093	(-0.242, 0.056)	0.219
Aridity index × Ecosystem (G)	-0.021	(-0.215, 0.174)	0.836
Drought duration × Aridity index × Ecosystem (G)	0.126	(0.016, 0.237)	**0.025**

Supplementary Table 6. Results of the multilevel meta-regression models for the effects of climate extreme indicators and their interactions with the aridity index, ecosystem type, community age, maximum designed species richness, and soil nutrients on the net biodiversity effect (a), complementarity effect (b), and selection effect (c) across experiments (*n* = 75). The following climate extreme indicators were used: maximum consecutive dry days defined using a 1-mm daily precipitation threshold (drought duration), number of days exceeding the 95th percentile of daily maximum temperature (extreme heat), and annual mean of the monthly standardized precipitation evapotranspiration index calculated using a 12-month integration period (water balance) within a given year. Each model initially included the main effects of climate extreme indicators and their interactions with ecosystem type and site-level environmental conditions (aridity index and soil PC1). Community age and maximum designed richness, along with their interactions with ecosystem type, were also included as covariates. Non-significant interactions ($P > 0.1$) were subsequently removed to obtain the final parsimonious models. Effect of ecosystem type represents the difference between grassland (G) and forest ecosystems, with forest serving as the reference level in the model. Soil nutrients were represented by the first principal component of soil properties (soil PC1; fig. S2). Each subtable reports all fixed-effect terms retained in the final parsimonious model. All main effects and prespecified covariates were retained, whereas non-significant interactions were removed during model simplification. β, meta-regression coefficient; CI, confidence interval. Significant *P* values ($P < 0.05$) are in bold. Analyses using the 12-month SPEI produced meta-

regression results nearly identical to those obtained with the 3-month SPEI used in the main models (Supplementary Table 2).

**(a) *Net biodiversity effect***			
Term	β	95% CI (lower-upper)	*P*
Drought duration	0.008	(-0.066, 0.081)	0.833
Extreme heat	0.053	(-0.004, 0.110)	0.066
Water balance	0.017	(-0.048, 0.081)	0.618
Aridity index	-0.041	(-0.151, 0.068)	0.458
Soil nutrients	0.012	(-0.085, 0.108)	0.816
Age	0.253	(0.173, 0.332)	**< 0.001**
Maximum designed richness	-0.258	(-0.364, -0.152)	**< 0.001**
Ecosystem (G)	0.638	(0.416, 0.860)	**< 0.001**

**(b) *Complementarity effect***			
Term	β	95% CI (lower-upper)	*P*
Drought duration	-0.068	(-0.209, 0.072)	0.340
Extreme heat	-0.003	(-0.056, 0.051)	0.928
Water balance	-0.032	(-0.094, 0.031)	0.325
Aridity index	-0.148	(-0.349, 0.053)	0.149
Soil nutrients	-0.039	(-0.154, 0.076)	0.506
Age	0.348	(0.274, 0.422)	**< 0.001**
Maximum designed richness	-0.201	(-0.308, -0.094)	**< 0.001**
Ecosystem (G)	0.698	(0.466, 0.930)	**< 0.001**
Drought duration × Aridity index	0.063	(-0.033, 0.158)	0.200

Drought duration × Ecosystem (G)	0.049	(-0.110, 0.208)	0.545
Aridity index × Ecosystem (G)	0.229	(-0.009, 0.468)	0.060
Drought duration × Aridity index × Ecosystem (G)	-0.140	(-0.258, -0.022)	**0.020**

(c) *Selection effect*

Term	β	95% CI (lower-upper)	*P*
Drought duration	0.119	(-0.009, 0.248)	0.069
Extreme heat	0.018	(-0.035, 0.071)	0.504
Water balance	0.014	(-0.050, 0.078)	0.663
Aridity index	0.027	(-0.129, 0.184)	0.732
Soil nutrients	-0.038	(-0.132, 0.055)	0.420
Age	-0.132	(-0.208, -0.056)	**< 0.001**
Maximum designed richness	-0.159	(-0.249, -0.070)	**< 0.001**
Ecosystem (G)	-0.019	(-0.213, 0.175)	0.846
Drought duration × Aridity index	-0.037	(-0.128, 0.054)	0.423
Drought duration × Ecosystem (G)	-0.093	(-0.242, 0.056)	0.222
Aridity index × Ecosystem (G)	-0.033	(-0.227, 0.160)	0.735
Drought duration × Aridity index × Ecosystem (G)	0.116	(0.004, 0.228)	**0.042**
