# Supplementary material for: Biodiversity effects under climate extremes intensify with aridity in grasslands but not forests
Source: Nat Ecol Evol. 2026 Jul 15;10(8):1464–74. doi: 10.1038/s41559-026-03130-1 (PMC13441892; doi:10.1038/s41559-026-03130-1)
Supplement: Supplementary file 2 [file 41559_2026_3130_MOESM2_ESM.pdf]

nature portfolio

Corresponding author(s): Takehiro Sasaki and Nico Eisenhauer

Last updated by author(s): May 19, 2026

Reporting Summary

Nature Portfolio wishes to improve the reproducibility of the work that we publish. This form provides structure for consistency and transparency in reporting. For further information on Nature Portfolio policies, see our Editorial Policies and the Editorial Policy Checklist.

Statistics

For all statistical analyses, confirm that the following items are present in the figure legend, table legend, main text, or Methods section.

n/a	Confirmed	
☐	☒	The exact sample size (*n*) for each experimental group/condition, given as a discrete number and unit of measurement
☐	☒	A statement on whether measurements were taken from distinct samples or whether the same sample was measured repeatedly
☐	☒	The statistical test(s) used AND whether they are one- or two-sided<br>*Only common tests should be described solely by name; describe more complex techniques in the Methods section.*
☐	☒	A description of all covariates tested
☐	☒	A description of any assumptions or corrections, such as tests of normality and adjustment for multiple comparisons
☐	☒	A full description of the statistical parameters including central tendency (e.g. means) or other basic estimates (e.g. regression coefficient) AND variation (e.g. standard deviation) or associated estimates of uncertainty (e.g. confidence intervals)
☐	☒	For null hypothesis testing, the test statistic (e.g. *F*, *t*, *r*) with confidence intervals, effect sizes, degrees of freedom and *P* value noted<br>*Give P values as exact values whenever suitable.*
☒	☐	For Bayesian analysis, information on the choice of priors and Markov chain Monte Carlo settings
☐	☒	For hierarchical and complex designs, identification of the appropriate level for tests and full reporting of outcomes
☐	☒	Estimates of effect sizes (e.g. Cohen's *d*, Pearson's *r*), indicating how they were calculated

Our web collection on statistics for biologists contains articles on many of the points above.

Software and code

Policy information about availability of computer code

Data collection	No software was used for data collection.
Data analysis	All analyses were conducted in R version 4.4.1, with meta-regressions fitted using the "metafor" package. The data and R code required to reproduce the results are are available in figshare (https://doi.org/10.6084/m9.figshare.30850508).

For manuscripts utilizing custom algorithms or software that are central to the research but not yet described in published literature, software must be made available to editors and reviewers. We strongly encourage code deposition in a community repository (e.g. GitHub). See the Nature Portfolio guidelines for submitting code & software for further information.

Data

Policy information about availability of data

All manuscripts must include a data availability statement. This statement should provide the following information, where applicable:

- Accession codes, unique identifiers, or web links for publicly available datasets
- A description of any restrictions on data availability
- For clinical datasets or third party data, please ensure that the statement adheres to our policy

The data and R code required to reproduce the results are available in figshare (https://doi.org/10.6084/m9.figshare.30850508). Daily precipitation and maximum temperature data are available from the Global Historical Climatology Network-Daily database (https://www.ncei.noaa.gov/products/land-based-station/global-historical-climatology-network-daily). Monthly SPEI is available from the SPEIbase v2.10 (https://developers.google.com/earth-engine/datasets/catalog/

CSIC_SPEI_2_10#terms-of-use). Monthly potential evapotranspiration is available from the Climatic Research Unit gridded Time Series 4.07 dataset (https://crudata.uea.ac.uk/cru/data/hrg/cru_ts_4.07/). Soil environmental data are available from the SoilGrids system (https://soilgrids.org/).

Research involving human participants, their data, or biological material

Policy information about studies with human participants or human data. See also policy information about sex, gender (identity/presentation), and sexual orientation and race, ethnicity and racism.

Reporting on sex and gender	n/a
Reporting on race, ethnicity, or other socially relevant groupings	n/a
Population characteristics	n/a
Recruitment	n/a
Ethics oversight	n/a

Note that full information on the approval of the study protocol must also be provided in the manuscript.

Field-specific reporting

Please select the one below that is the best fit for your research. If you are not sure, read the appropriate sections before making your selection.

☐ Life sciences ☐ Behavioural & social sciences ☒ Ecological, evolutionary & environmental sciences

For a reference copy of the document with all sections, see nature.com/documents/nr-reporting-summary-flat.pdf

Ecological, evolutionary & environmental sciences study design

All studies must disclose on these points even when the disclosure is negative.

Study description

We synthesized data from 75 biodiversity experiments across grasslands and forests to assess how aridity and soil nutrients modulate biodiversity effects under drought and heat extremes. We showed that biodiversity most strongly enhanced productivity under extreme drought in more-arid grasslands but had limited effects in forests under such conditions.

We assembled a global database by combining data from plant biodiversity experiments conducted in grasslands and forests. These experiments involved the manipulation of plant species richness and annual measurements of species-level aboveground plant biomass (in grasslands) or basal area (in forests) within each experimental plot. To be included, experiments had to meet the following criteria: (1) plant species richness was manipulated through sowing or planting; (2) experimental plots included both mixtures and corresponding monocultures to allow additive partitioning of biodiversity effects; (3) raw time-series data on species-level aboveground biomass or basal area were available; and (4) the experiment spanned a minimum of two years for grasslands, with measurements performed in at least two separate years, and at least three years for forests, with measurements performed in at least three separate years, allowing for the calculation of two points of annual basal area increments.

In each experiment, to maintain the target species composition and diversity, non-target species were typically removed from the plots on a repeated basis. For the forest experiments, we excluded data from the first two years following the initial planting because tree-planting experiments typically require a period of establishment, as suggested by a previous synthesis. In experiments that included additional treatments (e.g., fertilization), we used only plots with ambient conditions that were not subjected to those treatments. In total, we used data from 51 grassland and 24 forest biodiversity experiments conducted worldwide and spanning 2 to 23 years in duration (Fig. 1; Supplementary Table 1).

For each experiment, we first extracted data from plots with the maximum designed richness (i.e., the highest manipulated species richness treatment in that experiment) and derived key variables for each year, including the biomass or basal area of individual species and community age (years since the start of the experiment). Then, for each experiment and year, we calculated the mean and variance of the net biodiversity, complementarity, and selection effects. This approach minimized within-experiment variation associated with different richness levels and avoided pseudo-replication in the meta-regression analysis. The resulting meta-analytic dataset consisted of 340 independent data points, with 227 from grassland experiments and 113 from forest experiments.

Research sample

We compiled a database by combining original data on the aboveground productivity of each plant species across experimental plots from all experiments. For the grassland experiments, we used annual peak aboveground live biomass (g m^{-2}) as a measure of annual aboveground productivity for each species in each plot and year. These data were collected annually and consecutively across the grassland sites. For the forest experiments, we used annual basal area increment as an estimate of annual aboveground productivity for each species in each plot and year or multi-year period. Basal area (m^2 ha^{-1}) was calculated for each species using a standard equation based on the diameter at breast height (DBH). For experiments where DBH measurements were unavailable, we estimated the basal area according to the basal diameter measured at ground level.

Sampling strategy

For each experiment, we first extracted data from the original datasets for plots with the maximum designed richness (i.e., the

Sampling strategy	highest manipulated species-richness treatment in that experiment) and derived key variables for each year, including biomass or basal area of individual species and community age (years since the start of the experiment). The experiments included in this study prevented occasional immigration by repeatedly removing non-target species, thereby maintaining species composition and manipulative treatments.
Data collection	The resulting meta-analytic dataset consisted of 340 independent data points, with 227 from grassland experiments and 113 from forest experiments. For the grassland experiments, we used annual peak aboveground live biomass (g m^{-2}) as a measure of annual aboveground productivity for each species in each plot and year. These data were collected annually and consecutively across the grassland sites. For the forest experiments, we used annual basal area increment as an estimate of annual aboveground productivity for each species in each plot and year or multi-year period. Basal area (m^2 ha^{-1}) was calculated for each species using a standard equation based on the diameter at breast height (DBH). For experiments where DBH measurements were unavailable, we estimated the basal area according to the basal diameter measured at ground level.
Timing and spatial scale	For the grassland experiments, we used annual peak aboveground live biomass (g m^{-2}) as a measure of annual aboveground productivity for each species in each plot and year. For the forest experiments, we used annual basal area increment as an estimate of annual aboveground productivity for each species in each plot and year or multi-year period. Basal area (m^2 ha^{-1}) was calculated for each species using a standard equation based on the diameter at breast height (DBH). For experiments where DBH measurements were unavailable, we estimated the basal area according to the basal diameter measured at ground level. The plot size differed among experiments, ranging from4m^2 to 2025m^2. The dataset used in this study was collected from September 2024 to April 2025.
Data exclusions	Biodiversity experiments were included if they met the following criteria: (1) plant species richness was manipulated through sowing or planting; (2) plots included both species mixtures and corresponding monocultures, allowing additive partitioning of biodiversity effects; (3) raw time-series data on species-level aboveground biomass or basal area were available; and (4) experiments spanned at least two years for grasslands (with measurements in at least two separate years) and at least three years for forests (with measurements in at least three separate years), enabling calculation of two annual basal-area increments. In each experiment, to maintain the target species composition and diversity, non-target species were typically removed from the plots on a repeated basis. For the forest experiments, we excluded data from the first two years following the initial planting because tree-planting experiments typically require a period of establishment, as suggested by a previous synthesis. All available data were included in the analysis, with no data excluded.
Reproducibility	The data and R code required to reproduce the results are provided via a private link on figshare.
Randomization	This study did not implement additional randomization. In all original biodiversity experiments included, experimental plots were randomly assigned to species richness treatments at the time of experiment establishment.
Blinding	No blinding was applied during data acquisition or analysis, as this study used pre-existing experimental data and did not involve subjective outcome assessment.

Did the study involve field work? ☒ Yes ☐ No

Field work, collection and transport

Field conditions	We used data from 51 grassland and 24 forest biodiversity experiments conducted worldwide and spanning 2 to 23 years in duration. The experimental sites cover broad gradients of aridity and soil nutrient conditions. Annual climate anomalies were the main focus of our study, and we aimed to identify the environmental contexts under which biodiversity most strongly supports ecosystem productivity under climate extremes.
Location	The location of experimental sites is shown in Fig. 1a and Supplementary Table 1.
Access & import/export	Access to the study sites, as well as data collection and data sharing, by the principal investigators of each experiment followed standard practices and complied with all relevant local laws and regulations.
Disturbance	This study was a meta-level synthesis based on pre-existing biodiversity experiments and did not cause any additional disturbance to natural ecosystems. Any potential impacts associated with field data collection were minimized through the use of established walkways at each experimental site.

Reporting for specific materials, systems and methods

We require information from authors about some types of materials, experimental systems and methods used in many studies. Here, indicate whether each material, system or method listed is relevant to your study. If you are not sure if a list item applies to your research, read the appropriate section before selecting a response.

Materials & experimental systems

n/a	Involved in the study	
☒	☐	Antibodies
☒	☐	Eukaryotic cell lines
☒	☐	Palaeontology and archaeology
☒	☐	Animals and other organisms
☒	☐	Clinical data
☒	☐	Dual use research of concern
☐	☒	Plants

Methods

n/a	Involved in the study	
☒	☐	ChIP-seq
☒	☐	Flow cytometry
☒	☐	MRI-based neuroimaging

Dual use research of concern

Policy information about dual use research of concern

Hazards

Could the accidental, deliberate or reckless misuse of agents or technologies generated in the work, or the application of information presented in the manuscript, pose a threat to:

No	Yes	
☒	☐	Public health
☒	☐	National security
☒	☐	Crops and/or livestock
☒	☐	Ecosystems
☒	☐	Any other significant area

Experiments of concern

Does the work involve any of these experiments of concern:

No	Yes	
☒	☐	Demonstrate how to render a vaccine ineffective
☒	☐	Confer resistance to therapeutically useful antibiotics or antiviral agents
☒	☐	Enhance the virulence of a pathogen or render a nonpathogen virulent
☒	☐	Increase transmissibility of a pathogen
☒	☐	Alter the host range of a pathogen
☒	☐	Enable evasion of diagnostic/detection modalities
☒	☐	Enable the weaponization of a biological agent or toxin
☒	☐	Any other potentially harmful combination of experiments and agents

Plants

Seed stocks	Not applicable. This study is a synthesis of data from existing biodiversity experiments and did not generate, maintain, or distribute new seed stocks. Plant materials were originally sourced and managed within the individual contributing experiments.
Novel plant genotypes	Not applicable. No novel plant genotypes were generated or used in this study.
Authentication	Not applicable. No newly generated biological materials requiring authentication were produced in this study. Species identities and plant materials were based on those used in the original contributing biodiversity experiments.
