# Supplementary material for: Biodiversity effects under climate extremes intensify with aridity in grasslands but not forests
Source: Nat Ecol Evol. 2026 Jul 15;10(8):1464–74. doi: 10.1038/s41559-026-03130-1 (PMC13441892; doi:10.1038/s41559-026-03130-1)
Supplement: Supplementary file 3 [file 41559_2026_3130_MOESM3_ESM.pdf]

nature portfolio

# Peer Review File

# Biodiversity effects under climate extremes intensify with aridity in grasslands but not forests

Corresponding Author: Dr Takehiro Sasaki

Version 0:

Reviewer comments:

Reviewer #1

(Remarks to the Author)
This study compiles data from 75 biodiversity – functon(productivity) experiments conducted in grasslands and forests. It evaluates whether biodiversity effects (and their underlying mechanisms) are magnified under climatic extremes, and further whether these impacts are more important in already relatively arid conditions/locations. The study is well-designed, appropriately analysed and generally appropriately interpreted. The study is important and novel, making new strides in the evaluation of climate-related context dependency in biodiversity effects across systems. The finding that biodiversity effects underpinned by complementarity is most important in arid grassland systems under extended drought (but not heatwave) stress should really help to focus and prioritise the incorporation of biodiversity into restoration and management. The study also provides a timely reiteration of the importance of biodiversity for productivity across locations and ecosystems, with the compiled experimental data providing robust causal evidence and counteracting some recent challenges to this consensus based on re-analysis of observational data.

The authors note that the responsiveness of forests may be inherently lower than in grasslands, which may reduce the likelihood of detecting drought – diversity effect interactions. I agree. However, I would add that there may be several other factors that should be more explicitly considered. First, there is a substantially lower sample size and thus power for the forest vs. grassland studies. Second, as hinted at in the ms, drought may interact with biodiversity over long time periods, with a lag; for instance drought in one year may weaken herbivore defences with the impacts manifesting in the subsequent year. Drought impacts may also mainly manifest late in the growing season, with their impacts being more detectable in the subsequent year(s). Relatedly, growth may be a relatively weak indicator of drought – biodiversity interactions impacts in the growing season, with indicators of tree/crown health being potentially more sensitive. I would therefore urge the authors to more strongly caveat the largely null finding from forests and avoid heavy emphasis on the apparent contrast between the systems; this is especially important and currently lacking in the conclusion paragraph.

Linked to the comparisons between systems and the different findings, I would like to see some more critical discussion of the geographical and environmental distribution of experiments in the two systems. There is a conspicuous absence of grassland BEF experiments in Asia (despite there being experiments in China?), and particularly in the tropics; meanwhile there are a number of studies in tropical forests and perhaps fewer in more environmentally marginal (dry) sites. The authors should discuss and evaluate the potential impact on results and conclusions.

I also did not see how studies were found. Was this from the authors' network and knowledge alone, or were any systematic searches conducted? The inclusion criteria are clearly laid out, but what is missing is how studies were long-listed as potential candidates. In any meta-analysis one would expect this for full transparency and replicability.

Minor comments
340-346 – this negative effect of max diversity requires more explanation. I could understand this if NBE were standardised as per species (as in a slope) but they are just relative to the mean monoculture. The explanation implies that this variable was included after the others, but the model terms are fit simultaneously, so this doesn't seem to apply. This suggests there may be a genuinely weaker effect of biodiversity in richer systems.
Given caution and caveats around effects in forests and capacity to detect these, I think some more nuance and caution required in concluding statement.
In the methods the authors do not mention extraction of mono values, except mentioning calculation of the biodiversity effects – this stage perhaps worth mentioning?
It might be worth mentioning that selection effects can vary in space, such that diversity is important on larger scales than experimental plots, as some of the authors of this paper have previously demonstrated.

Reviewer #2

(Remarks to the Author)
The biodiversity-productivity relationship has long been a central pillar of community ecology and BEF research. Numerous experimental and observational studies have demonstrated that biodiversity enhances ecosystem productivity under average environmental conditions. However, whether these positive biodiversity effects persist, weaken, or intensify under climate extremes remains a major unresolved question, particularly in the context of accelerating droughts and heatwaves. This study provides one of the most comprehensive assessments to date of how environmental context modulates biodiversity effects under climate extremes by synthesizing data from 75 biodiversity experiments across grasslands and forests and integrating them with long-term climate records. The dataset is exceptionally valuable, the analytical framework is well grounded in BEF theory, and the separation of net biodiversity effects into complementarity and selection components adds important mechanistic insight. The finding that biodiversity most strongly enhances productivity under extreme drought in more-arid grasslands, primarily via strengthened complementarity effects, whereas forests show limited context dependence, is novel, timely, and potentially impactful. Overall, the manuscript makes a significant contribution to advancing context-dependent BEF theory and has clear implications for climate adaptation strategies.

However, I have three main suggestions that I believe would strengthen the manuscript and enhance the robustness and clarity of its conclusions.

1. The authors conclude that biodiversity most strongly enhances productivity under extreme drought in "more-arid grasslands." While this finding is intriguing, I would encourage greater caution in its generalization, particularly regarding the world's most arid grassland systems. Based on the metadata provided, grassland experiments are predominantly located in Europe and, to a lesser extent, North America. The annual precipitation range across grassland sites (533.7–1652.8 mm) indicates that most sites fall within mesic to moderately dry climates, with many concentrated in the 600–1000 mm interval. Truly arid and semi-arid grasslands (e.g., regions receiving <400 mm MAP, such as parts of the Mongolian Plateau or Tibetan Plateau) appear underrepresented. This raises an important question: would the observed patterns hold under more extreme aridity? Previous theoretical and empirical work (e.g., Baert et al. 2018, Ecology Letters) suggests that biodiversity–productivity relationships may exhibit non-linear responses along stress gradients, with biodiversity effects potentially weakening under extremely harsh conditions where strong abiotic filtering limits niche partitioning and facilitation. Under such circumstances, complementarity effects might be constrained rather than enhanced.
Therefore, I suggest the authors might consider supplementing their dataset with experiments from more arid grassland regions; or at minimum, the Discussion should acknowledge this geographic limitation and discuss the possibility that the relationship might not be linear across the full aridity gradient. The language throughout the manuscript could be appropriately qualified to avoid over-generalization beyond the climatic range represented in the current dataset.

2. The authors quantify dry conditions using annual aggregates (maximum consecutive dry days, frequency of extreme heat, annual mean SPEI). While this approach ensures comparability across sites, it may obscure ecologically important seasonal dynamics, particularly in grasslands where productivity is highly sensitive to the timing of drought relative to plant phenology. For example, early-growing-season drought often suppresses productivity more strongly than late-season drought, even if total drought duration is similar. By aggregating climate metrics annually, the analysis implicitly treats droughts occurring at different phenological stages as equivalent, which could influence estimates of biodiversity effects and their partitioning. If growing-season-specific metrics are available, incorporating them would strengthen ecological interpretation. If not, it would be helpful for the authors to acknowledge this limitation and discuss how seasonal asymmetry might influence their findings.
3. In the meta-regression models (Figure 2; Supplementary Tables), the main effects of Drought duration and Aridity index, as well as their two-way interaction, are not statistically significant. The significant result arises from the three-way interaction (Drought duration × Aridity index × Ecosystem type). While this indicates that the Drought duration × Aridity relationship differs between grasslands and forests, it does not directly demonstrate that the Drought duration × Aridity interaction is statistically significant within grasslands alone, the central claim of the manuscript. To strengthen interpretability and transparency, I recommend that the authors provide ecosystem-specific models (i.e., separate analyses for grasslands and forests) to directly test whether aridity significantly modulates drought effects on biodiversity responses within grasslands. Reporting effect sizes and confidence intervals for these models would further clarify the robustness of the core conclusion.

Decision Letter:

10th March 2026

*Please ensure you delete the link to your author homepage in this e-mail if you wish to forward it to your co-authors.

Dear Dr Sasaki,

Your manuscript entitled "Biodiversity effects under climate extremes intensify with aridity in grasslands but not forests" has now been assessed by two reviewers, whose reports you will find below. As you will see from their comments, the reviewers find the work of considerable potential interest but have raised a number of concerns to address. Therefore, we would like to see your responses to the comments along with a revised manuscript.

We are committed to providing a fair and constructive peer-review process. Do not hesitate to contact us if there are specific

requests from the reviewers that you believe are technically impossible or unlikely to yield a meaningful outcome.

When revising your manuscript:

* Please provide the manuscript in Microsoft Word format and highlight all changes in the same file or a copy.

* Include a “Response to reviewers” document detailing, point-by-point, how you addressed each reviewer comment. If no action was taken to address a point, you must provide a compelling argument. This response will be sent back to the reviewers along with the revised manuscript.

* If you have not done so already please begin to revise your manuscript so that it conforms to our Article format instructions at http://www.nature.com/natecolevol/info/final-submission. Refer also to any guidelines provided in this letter.

* Include a revised version of any required reporting checklist. It will be available to referees (and, potentially, statisticians) to aid in their evaluation if the manuscript goes back for peer review. A revised checklist is essential for re-review of the paper.

* Extended Data Figures - please ensure that any supplementary figures and tables that are crucial to the manuscript’s conclusions are converted into Extended Data figures and tables to increase visibility of these data. Extended Data figures and tables are online-only (present in the online PDF and full-text HTML versions of the paper), peer-reviewed display items that provide essential background to the article but are not included in the main article due to space constraints. A maximum of ten Extended Data display items (figures and tables) is permitted.

We hope to receive your revised manuscript within two months. If you cannot send it within this time, please let us know. We will be happy to consider your revision so long as nothing similar has been accepted for publication at Nature Ecology & Evolution or published elsewhere. Once ready, please use the link below to submit your revised manuscript and related files:

Link Redacted

**Note:** This URL links to your confidential home page and associated information about manuscripts you may have submitted, or that you are reviewing for us. If you wish to forward this email to co-authors, please delete the link to your homepage.

Nature Ecology & Evolution is committed to improving transparency in authorship. As part of our efforts in this direction, we are now requesting that all authors identified as ‘corresponding author’ on published papers create and link their Open Researcher and Contributor Identifier (ORCID) with their account on the Manuscript Tracking System (MTS), prior to acceptance. ORCID helps the scientific community achieve unambiguous attribution of all scholarly contributions. You can create and link your ORCID from the home page of the MTS by clicking on ‘Modify my Springer Nature account’. For more information please visit please visit <a href="http://www.springernature.com/orcid">www.springernature.com/orcid</a>.

Please do not hesitate to contact me if you have any questions or would like to discuss these revisions further.

[redacted]

**********************

Reviewers' comments:

Reviewer #1 (Remarks to the Author):

This study compiles data from 75 biodiversity – functon(productivity) experiments conducted in grasslands and forests. It evaluates whether biodiversity effects (and their underlying mechanisms) are magnified under climatic extremes, and further whether these impacts are more important in already relatively arid conditions/locations. The study is well-designed, appropriately analysed and generally appropriately interpreted. The study is important and novel, making new strides in the evaluation of climate-related context dependency in biodiversity effects across systems. The finding that biodiversity effects underpinned by complementarity is most important in arid grassland systems under extended drought (but not heatwave) stress should really help to focus and prioritise the incorporation of biodiversity into restoration and management. The study also provides a timely reiteration of the importance of biodiversity for productivity across locations and ecosystems, with the compiled experimental data providing robust causal evidence and counteracting some recent challenges to this consensus based on re-analysis of observational data.

The authors note that the responsiveness of forests may be inherently lower than in grasslands, which may reduce the likelihood of detecting drought – diversity effect interactions. I agree. However, I would add that there may be several other factors that should be more explicitly considered. First, there is a substantially lower sample size and thus power for the forest vs. grassland studies. Second, as hinted at in the ms, drought may interact with biodiversity over long time periods, with a lag; for instance drought in one year may weaken herbivore defences with the impacts manifesting in the subsequent

year. Drought impacts may also mainly manifest late in the growing season, with their impacts being more detectable in the subsequent year(s). Relatedly, growth may be a relatively weak indicator of drought – biodiversity interactions impacts in the growing season, with indicators of tree/crown health being potentially more sensitive. I would therefore urge the authors to more strongly caveat the largely null finding from forests and avoid heavy emphasis on the apparent contrast between the systems; this is especially important and currently lacking in the conclusion paragraph.

Linked to the comparisons between systems and the different findings, I would like to see some more critical discussion of the geographical and environmental distribution of experiments in the two systems. There is a conspicuous absence of grassland BEF experiments in Asia (despite there being experiments in China?), and particularly in the tropics; meanwhile there are a number of studies in tropical forests and perhaps fewer in more environmentally marginal (dry) sites. The authors should discuss and evaluate the potential impact on results and conclusions.

I also did not see how studies were found. Was this from the authors' network and knowledge alone, or were any systematic searches conducted? The inclusion criteria are clearly laid out, but what is missing is how studies were long-listed as potential candidates. In any meta-analysis one would expect this for full transparency and replicability.

Minor comments
340-346 – this negative effect of max diversity requires more explanation. I could understand this if NBE were standardised as per species (as in a slope) but they are just relative to the mean monoculture. The explanation implies that this variable was included after the others, but the model terms are fit simultaneously, so this doesn't seem to apply. This suggests there may be a genuinely weaker effect of biodiversity in richer systems.
Given caution and caveats around effects in forests and capacity to detect these, I think some more nuance and caution required in concluding statement.
In the methods the authors do not mention extraction of mono values, except mentioning calculation of the biodiversity effects – this stage perhaps worth mentioning?
It might be worth mentioning that selection effects can vary in space, such that diversity is important on larger scales than experimental plots, as some of the authors of this paper have previously demonstrated.

Reviewer #2 (Remarks to the Author):

The biodiversity-productivity relationship has long been a central pillar of community ecology and BEF research. Numerous experimental and observational studies have demonstrated that biodiversity enhances ecosystem productivity under average environmental conditions. However, whether these positive biodiversity effects persist, weaken, or intensify under climate extremes remains a major unresolved question, particularly in the context of accelerating droughts and heatwaves. This study provides one of the most comprehensive assessments to date of how environmental context modulates biodiversity effects under climate extremes by synthesizing data from 75 biodiversity experiments across grasslands and forests and integrating them with long-term climate records. The dataset is exceptionally valuable, the analytical framework is well grounded in BEF theory, and the separation of net biodiversity effects into complementarity and selection components adds important mechanistic insight. The finding that biodiversity most strongly enhances productivity under extreme drought in more-arid grasslands, primarily via strengthened complementarity effects, whereas forests show limited context dependence, is novel, timely, and potentially impactful. Overall, the manuscript makes a significant contribution to advancing context-dependent BEF theory and has clear implications for climate adaptation strategies.

However, I have three main suggestions that I believe would strengthen the manuscript and enhance the robustness and clarity of its conclusions.

1. The authors conclude that biodiversity most strongly enhances productivity under extreme drought in "more-arid grasslands." While this finding is intriguing, I would encourage greater caution in its generalization, particularly regarding the world's most arid grassland systems. Based on the metadata provided, grassland experiments are predominantly located in Europe and, to a lesser extent, North America. The annual precipitation range across grassland sites (533.7–1652.8 mm) indicates that most sites fall within mesic to moderately dry climates, with many concentrated in the 600–1000 mm interval. Truly arid and semi-arid grasslands (e.g., regions receiving <400 mm MAP, such as parts of the Mongolian Plateau or Tibetan Plateau) appear underrepresented. This raises an important question: would the observed patterns hold under more extreme aridity? Previous theoretical and empirical work (e.g., Baert et al. 2018, Ecology Letters) suggests that biodiversity–productivity relationships may exhibit non-linear responses along stress gradients, with biodiversity effects potentially weakening under extremely harsh conditions where strong abiotic filtering limits niche partitioning and facilitation. Under such circumstances, complementarity effects might be constrained rather than enhanced.
Therefore, I suggest the authors might consider supplementing their dataset with experiments from more arid grassland regions; or at minimum, the Discussion should acknowledge this geographic limitation and discuss the possibility that the relationship might not be linear across the full aridity gradient. The language throughout the manuscript could be appropriately qualified to avoid over-generalization beyond the climatic range represented in the current dataset.

2. The authors quantify dry conditions using annual aggregates (maximum consecutive dry days, frequency of extreme heat, annual mean SPEI). While this approach ensures comparability across sites, it may obscure ecologically important seasonal dynamics, particularly in grasslands where productivity is highly sensitive to the timing of drought relative to plant phenology. For example, early-growing-season drought often suppresses productivity more strongly than late-season drought, even if total drought duration is similar. By aggregating climate metrics annually, the analysis implicitly treats droughts occurring at

different phenological stages as equivalent, which could influence estimates of biodiversity effects and their partitioning. If growing-season-specific metrics are available, incorporating them would strengthen ecological interpretation. If not, it would be helpful for the authors to acknowledge this limitation and discuss how seasonal asymmetry might influence their findings.
3. In the meta-regression models (Figure 2; Supplementary Tables), the main effects of Drought duration and Aridity index, as well as their two-way interaction, are not statistically significant. The significant result arises from the three-way interaction (Drought duration × Aridity index × Ecosystem type). While this indicates that the Drought duration × Aridity relationship differs between grasslands and forests, it does not directly demonstrate that the Drought duration × Aridity interaction is statistically significant within grasslands alone, the central claim of the manuscript. To strengthen interpretability and transparency, I recommend that the authors provide ecosystem-specific models (i.e., separate analyses for grasslands and forests) to directly test whether aridity significantly modulates drought effects on biodiversity responses within grasslands. Reporting effect sizes and confidence intervals for these models would further clarify the robustness of the core conclusion.

Version 1:

Reviewer comments:

Reviewer #1

(Remarks to the Author)
The authors have thoroughly addressed comments made in the first round of review. The ms is now even stronger and clearer, including appropriate caveats, especially regarding the lack of aridity x diversity effects in forests. This impressive synthesis is poised to contribute substantially to the field and has important implications for our understanding of diversity's role across environmental stress gradients and ecosystems.

John Griffin

Reviewer #2

(Remarks to the Author)
I am pleased to see that the authors have addressed most of my previous suggestions and have made appropriate revisions and additions. Their efforts have effectively resolved my earlier concerns. In this round of review, I have no further questions.

Decision Letter:

6th May 2026

Dear Dr. Sasaki,

Thank you for submitting your revised manuscript "Biodiversity effects under climate extremes intensify with aridity in grasslands but not forests" (NATECOLEVOL-26010345A). Based on the reviewers' comments (appended below), we will be happy in principle to publish it in Nature Ecology & Evolution, pending minor revisions to satisfy some editorial requests and to comply with our formatting guidelines.

We are now performing detailed checks on your paper and will send you a checklist detailing our editorial and formatting requirements in about a week. Please do not upload the final materials and make any revisions until you receive this additional information from us.

Thank you again for your interest in Nature Ecology & Evolution. Please do not hesitate to contact me if you have any questions.

[redacted]

----------------

Reviewer #1 (Remarks to the Author):

The authors have thoroughly addressed comments made in the first round of review. The ms is now even stronger and clearer, including appropriate caveats, especially regarding the lack of aridity x diversity effects in forests. This impressive synthesis is poised to contribute substantially to the field and has important implications for our understanding of diversity's role across environmental stress gradients and ecosystems.

John Griffin

Reviewer #2 (Remarks to the Author):

I am pleased to see that the authors have addressed most of my previous suggestions and have made appropriate revisions and additions. Their efforts have effectively resolved my earlier concerns. In this round of review, I have no further questions.

**Open Access** This Peer Review File is licensed under a Creative Commons Attribution 4.0 International License, which permits use, sharing, adaptation, distribution and reproduction in any medium or format, as long as you give appropriate credit to the original author(s) and the source, provide a link to the Creative Commons license, and indicate if changes were made.
In cases where reviewers are anonymous, credit should be given to 'Anonymous Referee' and the source.
The images or other third party material in this Peer Review File are included in the article's Creative Commons license, unless indicated otherwise in a credit line to the material. If material is not included in the article's Creative Commons license and your intended use is not permitted by statutory regulation or exceeds the permitted use, you will need to obtain permission directly from the copyright holder.
To view a copy of this license, visit https://creativecommons.org/licenses/by/4.0/

<Responses and comments to Reviewer #1>

1. This study compiles data from 75 biodiversity–function(productivity) experiments conducted in grasslands and forests. It evaluates whether biodiversity effects (and their underlying mechanisms) are magnified under climatic extremes, and further whether these impacts are more important in already relatively arid conditions/locations. The study is well-designed, appropriately analysed and generally appropriately interpreted. The study is important and novel, making new strides in the evaluation of climate-related context dependency in biodiversity effects across systems. The finding that biodiversity effects underpinned by complementarity is most important in arid grassland systems under extended drought (but not heatwave) stress should really help to focus and prioritise the incorporation of biodiversity into restoration and management. The study also provides a timely reiteration of the importance of biodiversity for productivity across locations and ecosystems, with the compiled experimental data providing robust causal evidence and counteracting some recent challenges to this consensus based on re-analysis of observational data.

Response:

We appreciate your favorable comments on the manuscript. We have revised our paper in accordance with your suggestions and comments as described below.

2. The authors note that the responsiveness of forests may be inherently lower than in grasslands, which may reduce the likelihood of detecting drought – diversity effect interactions. I agree. However, I would add that there may be several other factors that should be more explicitly considered. First, there is a substantially lower sample size and thus power for the forest vs. grassland studies. Second, as hinted at in the ms, drought may interact with biodiversity over long time periods, with a lag; for instance drought in one year may weaken herbivore defences with the impacts manifesting in the subsequent year. Drought impacts may also mainly manifest late in the growing season, with their impacts being more detectable in the subsequent year(s). Relatedly, growth may be a relatively weak indicator of drought – biodiversity interactions impacts in the growing season, with indicators of tree/crown health being potentially more sensitive. I would therefore urge the authors to more strongly caveat the largely null finding from forests and avoid heavy emphasis on the apparent contrast between the systems; this is especially important and currently lacking in the conclusion paragraph.

Response:

Thank you for this helpful comment. We agree that the non-significant result for forests required clearer qualification. In response, we revised both the forest paragraph (Lines 297 to 299; Lines 304 to 306; Lines 306 to 313) and the concluding paragraph (Lines 366 to 368; Lines 380 to 382) to make this interpretation

more cautious. We now state explicitly that the forest dataset included fewer experiments than the grassland dataset and therefore had lower statistical power. We also clarify that slower forest demographic dynamics, possible multi-year lags in drought impacts, and the use of annual growth rather than more sensitive indicators of tree or crown condition may all have reduced our ability to detect effects of climatic anomalies on biodiversity effects in forests. Finally, we toned down the emphasis on a strong grassland–forest contrast in the conclusion and now note that this contrast should be viewed with caution. At the same time, we kept the message in the title, to represent the different findings for grasslands and forests.

3. Linked to the comparisons between systems and the different findings, I would like to see some more critical discussion of the geographical and environmental distribution of experiments in the two systems. There is a conspicuous absence of grassland BEF experiments in Asia (despite there being experiments in China?), and particularly in the tropics; meanwhile there are a number of studies in tropical forests and perhaps fewer in more environmentally marginal (dry) sites. The authors should discuss and evaluate the potential impact on results and conclusions.

Response:
Thank you for this valuable comment. We agree that the geographic and environmental distribution of available experiments is uneven and may influence comparisons between grasslands and forests. We have therefore revised the Discussion to more explicitly acknowledge that grassland experiments are concentrated mainly in Europe and North America, with sparse or absent representation from Asian grasslands, tropical grasslands, dry tropical forests, and Southern Hemisphere systems, whereas forest experiments include more tropical sites but fewer environmentally marginal dry sites. We now state more clearly that these imbalances constrain the scope of inference of our synthesis and highlight the need for new BEF experiments in underrepresented regions (Lines 372 to 380). In doing so, we also addressed the related concern raised by Reviewer 2 by qualifying our conclusions about aridity effects to the climatic range represented in the current dataset.

4. I also did not see how studies were found. Was this from the authors' network and knowledge alone, or were any systematic searches conducted? The inclusion criteria are clearly laid out, but what is missing is how studies were long-listed as potential candidates. In any meta-analysis one would expect this for full transparency and replicability.

Response:
Thank you for this helpful comment. We agree that the original manuscript did not sufficiently explain how candidate studies were identified. This synthesis was not based on published summary statistics alone.

Instead, it required raw data on monoculture and mixture productivity from repeated measurements through time, both to enable additive partitioning of biodiversity effects and to match their responses to year-to-year climate extreme indicators over the corresponding observation intervals. Because these data are often not fully available in published articles, we assembled candidate datasets from the authors' knowledge of global grassland and forest BEF experiments, previous BEF syntheses, and associated research networks, and then screened them against the predefined inclusion criteria. We have now revised the Methods to describe this study-identification procedure explicitly (Lines 611 to 617). The reproducibility of the final dataset is further supported by the explicit inclusion criteria and by Supplementary Table 1, which reports the included experiments, sites, durations, and the highest manipulated species richness treatment in each experiment.

5. Minor comments. 340-346 – this negative effect of max diversity requires more explanation. I could understand this if NBE were standardised as per species (as in a slope) but they are just relative to the mean monoculture. The explanation implies that this variable was included after the others, but the model terms are fit simultaneously, so this doesn't seem to apply. This suggests there may be a genuinely weaker effect of biodiversity in richer systems.

Response:

Thank you for raising this point. We agree that our previous explanation of the negative coefficients of maximum designed richness was not sufficiently clear, and that the sentence implying a sequential interpretation of model terms was inappropriate. We have therefore revised this passage (Lines 354 to 363). We now clarify that, because our meta-analysis used only the highest-diversity treatment from each experiment, maximum designed richness functions as an experiment-level design covariate describing the upper bound of richness manipulation, rather than a within-experiment test of richness effects. Accordingly, we do not interpret the negative coefficients as evidence that biodiversity effects are intrinsically weaker in richer systems, but rather as reflecting among-experiment differences associated with richness design and cross-experiment standardization. This is now better represented in the revised version of our manuscript.

6. Given caution and caveats around effects in forests and capacity to detect these, I think some more nuance and caution required in concluding statement.

Response:

Thank you for this suggestion. We agree and have revised the forest paragraph and concluding paragraph accordingly. As detailed in our response to Comment 3, we now state more explicitly that the forest result should be interpreted cautiously, because the forest dataset included fewer experiments, was temporally coarser, and may be less able to capture delayed drought effects.

YOKOHAMA National University
79-1 Tokiwadai, Hodogaya-ku, Yokohama 240-8501 JAPAN

7. In the methods the authors do not mention extraction of mono values, except mentioning calculation of the biodiversity effects – this stage perhaps worth mentioning?

Response:
Thank you for the suggestion. We agree and have now clarified in the Methods how monoculture values were extracted for the additive partitioning, including matching by experiment and year (or observation interval in forests) and, where relevant, by block (Lines 667 to 671).

8. It might be worth mentioning that selection effects can vary in space, such that diversity is important on larger scales than experimental plots, as some of the authors of this paper have previously demonstrated.

Response:
Thank you for this helpful comment. We agree and have now added a sentence to the opening paragraph of the Results and Discussion noting that variation in selection effects among experiments may imply that biodiversity contributes to ecosystem functioning at broader spatial scales through spatial turnover in species performance (Lines 247 to 250).

<Responses and comments to Reviewer #2>
9. The biodiversity-productivity relationship has long been a central pillar of community ecology and BEF research. Numerous experimental and observational studies have demonstrated that biodiversity enhances ecosystem productivity under average environmental conditions. However, whether these positive biodiversity effects persist, weaken, or intensify under climate extremes remains a major unresolved question, particularly in the context of accelerating droughts and heatwaves. This study provides one of the most comprehensive assessments to date of how environmental context modulates biodiversity effects under climate extremes by synthesizing data from 75 biodiversity experiments across grasslands and forests and integrating them with long-term climate records. The dataset is exceptionally valuable, the analytical framework is well grounded in BEF theory, and the separation of net biodiversity effects into complementarity and selection components adds important mechanistic insight. The finding that biodiversity most strongly enhances productivity under extreme drought in more-arid grasslands, primarily via strengthened complementarity effects, whereas forests show limited context dependence, is novel, timely, and potentially impactful. Overall, the manuscript makes a significant contribution to advancing context-dependent BEF theory and has clear implications for climate adaptation strategies.

Response:

Thank you for your thoughtful and encouraging comment. We have revised our paper in accordance with your suggestions and comments as described below.

10. However, I have three main suggestions that I believe would strengthen the manuscript and enhance the robustness and clarity of its conclusions. The authors conclude that biodiversity most strongly enhances productivity under extreme drought in "more-arid grasslands." While this finding is intriguing, I would encourage greater caution in its generalization, particularly regarding the world's most arid grassland systems. Based on the metadata provided, grassland experiments are predominantly located in Europe and, to a lesser extent, North America. The annual precipitation range across grassland sites (533.7–1652.8 mm) indicates that most sites fall within mesic to moderately dry climates, with many concentrated in the 600–1000 mm interval. Truly arid and semi-arid grasslands (e.g., regions receiving <400 mm MAP, such as parts of the Mongolian Plateau or Tibetan Plateau) appear underrepresented. This raises an important question: would the observed patterns hold under more extreme aridity? Previous theoretical and empirical work (e.g., Baert et al. 2018, Ecology Letters) suggests that biodiversity–productivity relationships may exhibit non-linear responses along stress gradients, with biodiversity effects potentially weakening under extremely harsh conditions where strong abiotic filtering limits niche partitioning and facilitation. Under such circumstances, complementarity effects might be constrained rather than enhanced. Therefore, I suggest the authors might consider supplementing their dataset with experiments from more arid grassland regions; or at minimum, the Discussion should acknowledge this geographic limitation and discuss the possibility that the relationship might not be linear across the full aridity gradient. The language throughout the manuscript could be appropriately qualified to avoid over-generalization beyond the climatic range represented in the current dataset.

Response:
Thank you for this insightful comment. We agree that our grassland dataset does not fully capture the most arid grassland systems and therefore does not allow us to infer whether the observed pattern would persist under more extreme aridity. We have revised the Discussion to state this limitation explicitly, to note that the most arid grasslands are underrepresented, and to acknowledge that biodiversity effects may respond non-linearly at higher aridity, potentially weakening where strong abiotic filtering constrains complementarity (Lines 372 to 380). We have also qualified the language used throughout the manuscript to avoid over-generalization beyond the climatic range represented by the current dataset. As also suggested by Reviewer 1, we now more clearly discuss the geographic bias in site distribution and the need for new BEF experiments in underrepresented dry regions. We were not able to expand the dataset further because, to our knowledge, no additional experiments from these regions currently meet our inclusion criteria, particularly with respect to sown diversity gradients and data suitable for additive partitioning of biodiversity

YOKOHAMA National University
79-1 Tokiwadai, Hodogaya-ku, Yokohama 240-8501 JAPAN

effects.

11. The authors quantify dry conditions using annual aggregates (maximum consecutive dry days, frequency of extreme heat, annual mean SPEI). While this approach ensures comparability across sites, it may obscure ecologically important seasonal dynamics, particularly in grasslands where productivity is highly sensitive to the timing of drought relative to plant phenology. For example, early-growing-season drought often suppresses productivity more strongly than late-season drought, even if total drought duration is similar. By aggregating climate metrics annually, the analysis implicitly treats droughts occurring at different phenological stages as equivalent, which could influence estimates of biodiversity effects and their partitioning. If growing-season-specific metrics are available, incorporating them would strengthen ecological interpretation. If not, it would be helpful for the authors to acknowledge this limitation and discuss how seasonal asymmetry might influence their findings.

Response:
Thank you for this valuable comment. We agree that the timing of climatic extremes relative to plant phenology can influence productivity responses, particularly in grasslands. To address this concern, we recalculated maximum consecutive dry days, extreme heat frequency, and SPEI using warm-season conditions (April–September for all non-tropical sites, which were exclusively in the Northern Hemisphere, and year-round in tropical regions) and repeated the meta-regression analyses using these alternative metrics. These supplementary analyses did not detect any context-dependent effects of warm-season climate extremes on net biodiversity, complementarity, or selection effects (Supplementary Table 3). We have therefore added this sensitivity analysis to the Methods (Lines 750 to 755) and Supplementary Information (Supplementary Table 3), and now note in the Results and Discussion that biodiversity responses may integrate climatic stress over annual timescales rather than being driven solely by within-season extremes (Lines 284 to 288).

12. In the meta-regression models (Figure 2; Supplementary Tables), the main effects of Drought duration and Aridity index, as well as their two-way interaction, are not statistically significant. The significant result arises from the three-way interaction (Drought duration × Aridity index × Ecosystem type). While this indicates that the Drought duration × Aridity relationship differs between grasslands and forests, it does not directly demonstrate that the Drought duration × Aridity interaction is statistically significant within grasslands alone, the central claim of the manuscript. To strengthen interpretability and transparency, I recommend that the authors provide ecosystem-specific models (i.e., separate analyses for grasslands and forests) to directly test whether aridity significantly modulates drought effects on biodiversity responses within grasslands. Reporting effect sizes and confidence intervals for these models would further clarify the

robustness of the core conclusion.

Response:

Thank you for this helpful comment. Following this recommendation, we directly tested ecosystem-specific slopes of the maximum consecutive dry days × aridity index interaction within the full meta-regression model using post-hoc linear contrasts. This analysis showed that the drought duration × aridity interaction was significant in grasslands ($\beta = -0.07$, 95% CI = [−0.14, −0.01], $P = 0.03$), but not in forests ($\beta = 0.06$, 95% CI = [−0.03, 0.16], $P = 0.20$). These results confirm that aridity significantly modulates drought effects on complementarity within grasslands, consistent with our conclusion. We have now added these ecosystem-specific slope estimates to the Results and Discussion (Lines 263 to 266) and clarified in the Methods that significant three-way interactions were followed by post hoc simple-slope tests using linear contrasts within the fitted meta-regression models (Lines 794 to 797).

YOKOHAMA National University
79-1 Tokiwadai, Hodogaya-ku, Yokohama 240-8501 JAPAN
